# CRF-like receptor SEB-3 in sex-common interneurons potentiates stress handling and reproductive drive in *C. elegans*

**DOI:** 10.1038/ncomms11957

**Published:** 2016-06-20

**Authors:** Changhoon Jee, Jimmy F. Goncalves, Brigitte LeBoeuf, L. Rene Garcia

**Affiliations:** 1 Department of Biology, Howard Hughes Medical Institute, Texas A&M University, 3258 TAMU, College Station, Texas 77843-3258, USA

## Abstract

Environmental conditions can modulate innate behaviours. Although male *Caenorhabditis elegans* copulation can be perturbed in the presence of stress, the mechanisms underlying its decision to sustain copulation are unclear. Here we describe a mating interference assay, which quantifies the persistence of male *C. elegans* copulation in noxious blue light. We show that between copulations, the male escapes from blue light illumination at intensities over 370 μW mm^−2^. This response is attenuated in mutants with constitutive activation of the corticotropin-releasing factor receptor family homologue SEB-3. We show that activation of this receptor causes sex-common glutamatergic lumbar ganglion interneurons (LUA) to potentiate downstream male-specific reproduction circuits, allowing copulatory behaviours to partially override the light-induced escape responses in the male. SEB-3 activation in LUA also potentiates copulation during mild starvation. We suggest that SEB-3 activation allows *C. elegans* to acclimate to the environment and thus continue to execute innate behaviours even under non-optimal conditions.

## Abstract (web summary)

Innate animal behaviours can be negatively regulated by environmental stressors. Jee *et al.* show that suppression of male *C. elegans* copulation behaviour by noxious light can be overcome by activation of SEB-3, a homologue of the stress-associated mammalian corticotropin-releasing factor receptor family.

## Graphical abstract

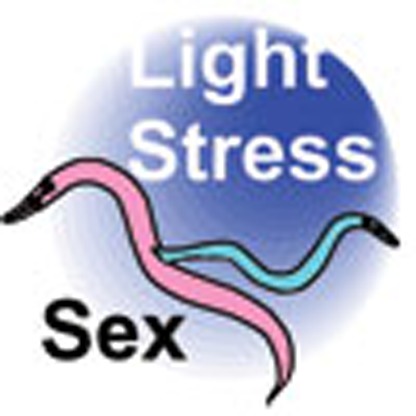

The strength of an animal's physiological need such as food acquisition or reproduction regulates the execution of innate behaviours required to satisfy those motivated compulsions. However, the animal must also monitor its environment and determine whether stressful conditions warrant the expression of their innate urges. In the vertebrate brain, neurons in the hypothalamic, pituitary and adrenal regions (the HPA axis) monitor the animal's metabolic, homeostatic and reproductive state, and subsequently produce peptides and neurotransmitters that direct the appropriate behaviour^1^,^2^,^3^. Accumulated research has suggested that in the HPA axis and non-hypothalamic regions, the corticotropin-releasing factor (CRF) peptide family mediates how an animal executes innate and learned behaviours under stress conditions^4^,^5^,^6^.

The CRF peptide family includes the molecules CRF, urocortin 1, urocortin 2 and urocortin 3 (refs 7, 8). These peptides transduce their activity through the CRF1 and CRF2 G-protein-coupled receptors^9^,^10^,^11^. Much of our understanding about the CRF receptor and its peptides comes from rodent studies using ethanol and controlled substances as neurochemical surrogates of endogenous re-inforcers^12^,^13^,^14^. The current idea is that CRF1 and CRF2 receptors in non-hypothalamic neurons can modulate the level of increased behavioural arousal and motivated behaviours in response to external stress^15^,^16^,^17^. However, whether the animal uses activated CRF receptors as an alert mechanism to escape from, or to cope and function in, stressful environments might depend on the context and intensity of the stress.

Clarification of how animals sustain innate behaviours in a continuum of environmental insults can be addressed with an organism containing a compact, well-described nervous system, such as *C. elegans*. The CRF system is conserved in the nematode^18^. *C. elegans* contains a CRF receptor family-like G-protein coupled receptor (GPCR), SEB-3 (SEcretin/class B GPCR). Similar to the mammalian CRFR1 receptor, SEB-3 also promotes arousal and mediates appropriate behavioural responses to noxious stimuli^18^. Genetic gain- and loss-of-function *seb-3* alleles have been isolated in *C. elegans*, which allows one to address how the intensity of behaviours, used to satisfy urges, can change in stress conditions^18^.

*C. elegans* copulation is a motivated behaviour that can be dissected through experimentation. This innate behaviour is regulated by the male's neural-muscular circuitry, metabolic state and his innate reproductive urge^19^,^20^,^21^,^22^,^23^,^24^,^25^. A well-fed male will explore the environment and even leave a food source to search for mates. When the male contacts a hermaphrodite, it stops mate searching, presses its body against his mate's cuticle and crawls backwards around its body. The male will circle its mate until it locates and then intromit his copulatory spicules in the vulva. As the male executes these behaviours, it must maintain its sexual drive until it ejaculates.

Here we asked what factors modulate the male's tenacity to copulate in intensifying external stress. We developed a mating interference (MI) assay that uses 475-nm (blue) light as a stressor. Crawling males sense blue light as a noxious stimulus and avoid it^26^,^27^,^28^. In contrast, we found that SEB-3 promotes increased activity in the LUA interneurons, to allow males *in copula* to tolerate moderate intensities of blue light. Our findings suggest that the male uses the CRF receptor family-like GPCR to sustain innate motivated behaviours in moderate stress.

## Results

### Noxious stimuli interfere with copulation behavior

**Figure 1:**
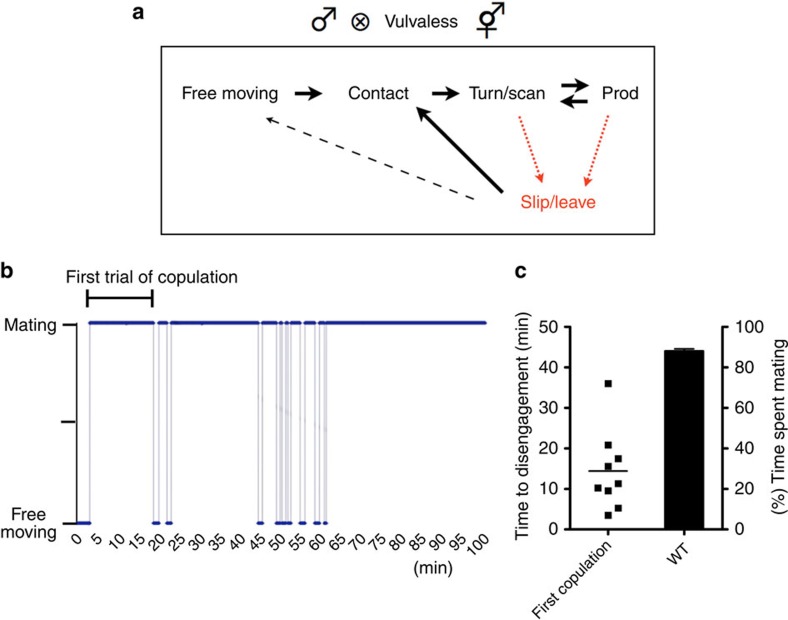
Persistence of male mating with a vulvaless hermaphrodite. A virgin WT male was paired with a vulvaless, static hermaphrodite on a NGM plate with *E. coli* as a food source. The males' activity was recorded for 100 min. (**a**) Summary of the behavioural transitions of a male mating with a vulvaless hermaphrodite. The male initiates copulation when its tail contacts the hermaphrodite. The male moves backwards along the hermaphrodite's cuticle, while it scans for the vulva. When the tail reaches the end of the hermaphrodite' body, the male turns to the other and continues backward. When turning at the ends of the hermaphrodite or scanning along its cuticle, the male would disengage from its partner. Shortly, it returns to the hermaphrodite and reinitiate copulation behaviour as shown in **b**. (**b**) Copulatory behaviour of a single male with a vulvaless static hermaphrodite was recorded for 100 min. ‘Mating' indicates the duration from contact to disengagement. (**c**) The left *y* axis represents the duration of the first copulatory act as indicated in **b** and the right *y* axis displays the amount of time a WT male spends copulating without extinction (*n*=9). Bar represents mean on the left and the error bar represents s.d. on the right.

To study how *C. elegans* male sexual drive is regulated, we established an assay to quantify the tenacity of the male to copulate under unfavourable conditions. When mated with a non-moving, easily penetrable mate, *C. elegans* male copulation behaviour can be completed within 2 min^29^. To determine the duration a male can sustain futile copulation attempts, we paired a virgin male with vulvaless, non-moving hermaphrodites and measured how long until the male stops searching for the non-existent vulva. Although the male searched for the non-existent vulva, its locomotor behaviour switched between bouts of backward locomotion and short pauses, during which it would prod the hermaphrodite's cuticle with its spicules. When turning at the ends of the hermaphrodite or scanning along its cuticle, the male would lose contact with his partner, which we term disengagement. However, the male would return and reinitiate scanning (Fig. 1a,b). During the first copulation attempt, the males maintained contact for 2–36 min, with an average time of 14.42 min (s.d. ±9.820) until the first disengagement (Fig. 1c). The times between mating re-attempts were short and the males spent 87% of their 100 min engaged in copulation (Fig. 1b,c).

**Figure 2:**
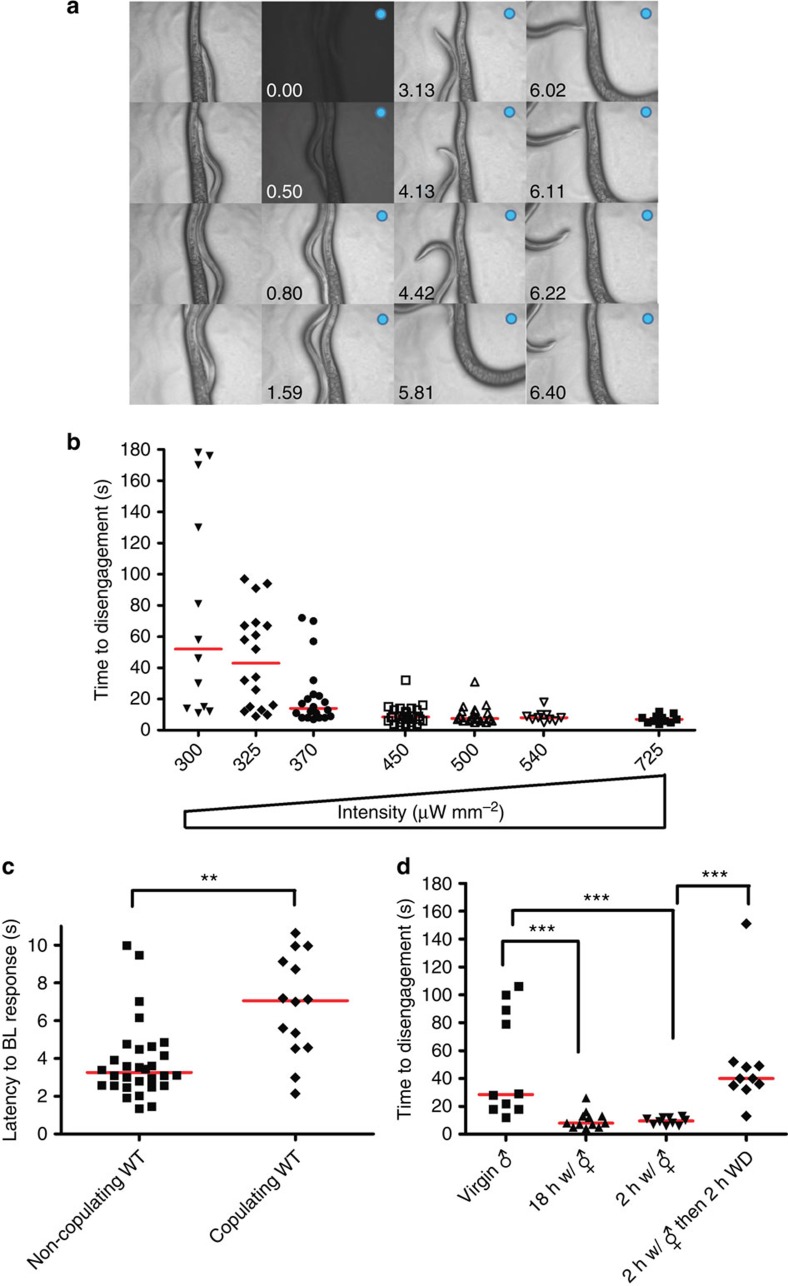
The MI assay measures motivation. (**a**) Steps in the MI assay under 725 μW mm^−2^ blue light. The blue dot denotes activation of blue light and the time is specified. The worm in the centre of each image is the hermaphrodite; neither its head nor its tail are visible. In contrast, the smaller male moves around in the different images; the male's tail is indicated by a closed arrow and the male's head is indicated by an open arrow. (**b**) Behavioural quantification of copulation tenacity against the blue light irritant. The *x* axis indicates the blue light intensity used. Thw *y* axis indicates the amount of time it took a copulating male to disengage from a hermaphrodite after the blue light had been turned on. *N*=12, 18, 20, 24, 20, 10 and 11 (from left to right in order of each group). (**c**) The latency to blue light (450 μW mm^−2^) aversion is attenuated in copulating males. The *x* axis indicates the behavioural state of the male before blue light activation. The *y* axis indicates the time from blue light activation to first directional change, even if in the case of mating males, they remain at the hermaphrodite. *N* number is 30 (non-copulating WT) and 14 (copulating WT). (**d**) The MI assay on non-virgin males using 370 μW mm^−2^ blue light. The *x* axis indicates the amount of time a male was incubated with a hermaphrodite (WD; withdrawal from hermaphrodite). *N*=10, 12, 10 and 10 (from left to right in order of each group). The *y* axis indicates the time it took a male to leave its partner. Mann–Whitney test indicated; **P<0.01, ***P<0.001.

We then asked how long the male could maintain futile copulation behaviour, if it was simultaneously exposed to an external irritant. To address this question, the MI assay was developed, using 475-nm (blue) light to interfere with the male's mating behaviour. Blue light is an aversive stimulus, which hermaphrodite worms avoid^26^,^27^. We found that high intensity (725 μW mm^−2^) blue light also disturbs mating behaviour and causes males to disengage from copulation within 7 s (Fig. 2a). When the male is exposed to 725 μW mm^−2^ blue light, it hesitates between crawling forward to escape the light and backward locomotion to resume scanning for the vulva. Eventually, the male will disengage from his partner. Thus, although the male is still interested in mating, its aversive response to noxious blue light will override the mating drive. This suggests that blue light-induced MI can be used to determine mechanisms that regulate a goal-oriented motivated behaviour.

We hypothesized that the male's endurance to copulate in varying intensities of 475-nm (blue) light might be proportional to his drive to mate. For example, the lower its sex drive, the sooner that blue light can induce him to disengage. To explore this possibility, we first quantified what range of blue light intensities a virgin male can tolerate before it disengages from copulation (Fig. 2b). Light intensities ranging from 725 to 450 μW mm^−2^ caused copulating virgins to disengage from their partners in <20 s. At 450 μW mm^−2^, a copulating male will endure the irradiation longer than if it was roaming (Fig. 2c). However, when irradiated with lower light intensities (370–300 μW mm^−2^), males copulated for longer periods with an intensity-dependent trend (Fig. 2b).

To verify that the copulating male was responding to 475-nm (blue) light and not to heat, we used 500 μW mm^−2^ irradiation to test virgin males that lacked the *lite-1* receptor. LITE-1 is a *C. elegans* gustatory-like receptor that is required for light-repulsion^26^,^28^,^30^. The MI assay was conducted with wild type (WT; *n*=10) and *lite-1* virgins (*n*=10). In contrast to the WT, 100% of *lite-1* males maintained copulation behaviour for >60 s in the blue light (Fisher's exact test *P*<0.0001). Thus, the WT virgins are negatively responding to the blue light.

Next we asked whether blue light tolerance is correlated with the male's motivational drive to mate. Previous work has shown that repeated mating reduces subsequent mating drive in males^29^. Males were either kept secluded from hermaphrodites for 18 h or allowed to mate *ad libitum* for 2 or 18 h with vulva-containing hermaphrodites. On plates that contained hermaphrodites, any male not engaged in copulation was then used in the MI assay. Males that were allowed to mate *ad libitum* responded more negatively to blue light by disengaging from their mating partners faster than virgins (Fig. 2d). Thus, post-coital males are less tolerant to blue light after mating. We then asked how long after copulation could blue light tolerance be restored. Prior work showed that after ejaculation, ∼12 min refractory period is necessary for males to regain some of their mating drive and to reattempt mating^29^; however, the time required for full recovery of their mating drive was not known. We determined that 2 h of sexual deprivation was sufficient to restore blue light toleration (Fig. 2d). Thus, these results suggest that the MI assay can be used as a proxy measure for the male's sexual drive state.

### SEB-3 facilitates males drive to copulate in noxious stimuli

**Figure 3:**
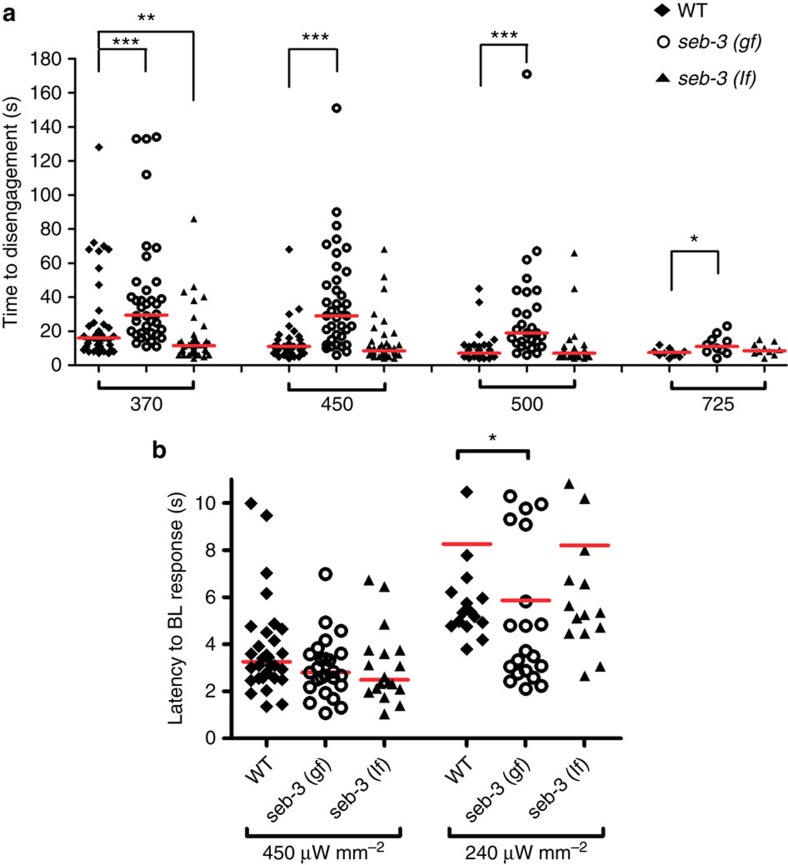
Activation of SEB-3 results in enhanced motivation. (**a**) Quantification of *seb-3* mutants' copulation tenacity using the MI assay. Tenacity of copulation is represented by the time of disengagement (*y* axis) under different intensities of blue light irritant (μW mm^−2^) (*x* axis). *N*=39, 38, 39, 40, 37, 40, 30, 30, 30, 10, 10 and 10 (from left to right in order of each group). **P<0.01 and ***P<0.001 (Mann–Whitney test). (**b**) Non-copulating *seb-3* mutant males avoid blue light, similar to WT males. The response to blue light (450 μW mm^−2^) was determined when the crawling male stops its movement and changes direction to escape the illuminated area (*y* axis). *N*=30, 25, 17, 20, 22 and 20 (from left to right in order of each group). *P<0.05 (Mann–Whitney test).

In mammals, activation of neural stress systems reinforce motivated behaviours and misregulation of these systems leads to animals performing behaviours under inappropriate circumstances^31^. To identify what modulates the the male's sexual intensity, we quantified how copulating *seb-3* mutant males respond in the MI assay. SEB-3, a CRF receptor-like GPCR, mediates stress responses in *C. elegans*. The gain-of-function *seb-3* (*gf*) allele provides an *in vivo* model of a constitutively activated stress response system^18^. Under standard conditions, we found *seb-3 (gf)* males spent more time copulating with vulvaless mates, whereas the opposite was observed for the *seb-3 (lf)* male (Supplementary Fig. 1a); however, during their first copulation, both mutants engage their mates longer than WT (Supplementary Fig. 1b). In contrast, we found that the copulating *seb-3(gf)* males endure more 475-nm (blue) light at all intensities, whereas the *seb-3* deletion mutant is less tolerant than WT to even moderate intensities of blue light while copulating (Fig. 3a). Interestingly, if males are not mating, the *seb-3(gf)* and *(lf)* males avoid 450 μW mm^−2^ light, similar to WT (Fig. 3b). At lower intensity (240 μW mm^−2^), *seb-3 (gf)* males responded faster than WT or *seb-3 (lf)* males (Fig. 3b), indicating that the allele does not make the males insensitive to 475-nm light. This result suggests the *seb-3* mutations do not affect the males' response to light, but rather modulates their sexual drive, so that copulation behaviour takes precedence over mild aversive stress.

**Figure 4:**
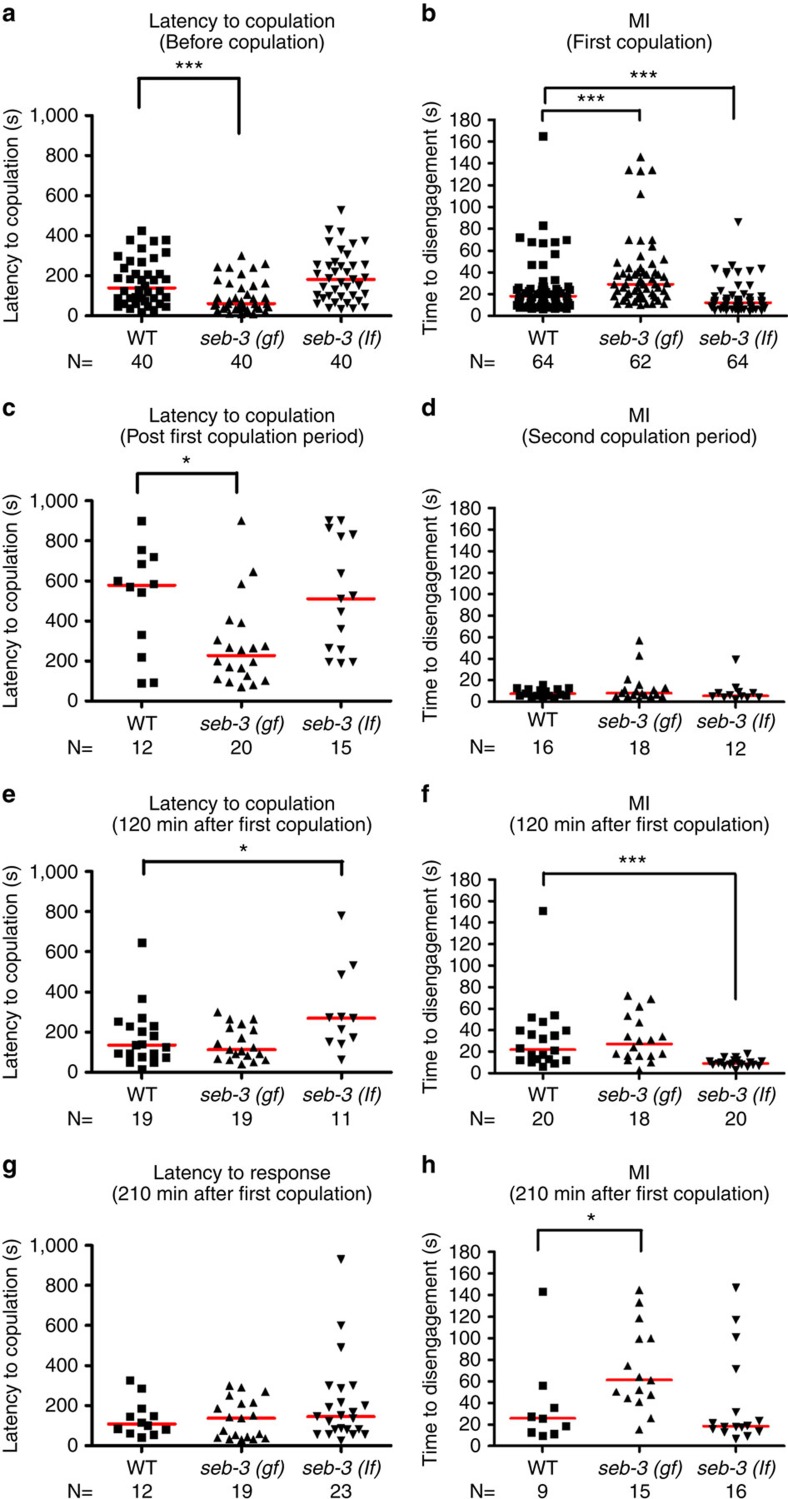
SEB-3 reinforces the mating drive of males. (**a**,**c**,**e**,**g**) Latency to initiate copulation before blue light exposure. (**b**,**d**,**f**,**h**) The MI assay was conducted under 370 μW mm^−2^ intensity of blue light exposure. The latency to copulate and time to disengagement in MI assay are shown for copulation-deprived males (**a**,**b**), postcoital males (**c**,**d**), males at 2 h recovery post copulation (**e**,**f**) and males at 3 h 30 min recovery post copulation (**g**,**h**). *P<0.05, **P<0.01 and ***P<0.001 (Mann–Whitney test).

We next addressed whether SEB-3 reinforces sexual drive under conditions such as (i) mating deprivation, (ii) immediate sexual recovery post copulation and (iii) 2–3.5 h recovery after copulation. Consistent with the idea that SEB-3 potentiates sex drive, we found that the 24-h-old virgin *seb-3(gf)* male initiated mating behaviour faster than the WT (Fig. 4a) and they displayed increased tolerance to 475-nm (blue) light (Fig. 4b). In contrast to *seb-3(gf)* males, virgin *seb-3(lf)* males displayed less tolerance to blue light (Fig. 4b). Similar to the WT, post-coital *seb-3(gf)* and *(lf)* males took longer than virgins to re-attempt mating and they also showed reduced tolerance to blue light (Fig. 4c,d). However, the post-coital *seb-3(gf)* male re-attempted copulation faster than WT (Fig. 4c). Under conditions where the post-coital males were segregated from hermaphrodites for 2 h, *seb-3(lf)* males still required a longer time to re-initiate mating and were less tolerant to blue light than WT and *seb-3(gf)* males (Fig. 4e,f). After 3.5 h of segregation from hermaphrodites, *seb-3(lf)* post-coital males displayed blue light tolerance that was similar to virgins (Fig. 4g,h). Taken together, the data suggest that SEB-3 functions in reinforcing the male's sexual drive.

### The LUA neurons express SEB-3 and enhance male sexual drive

**Figure 5:**
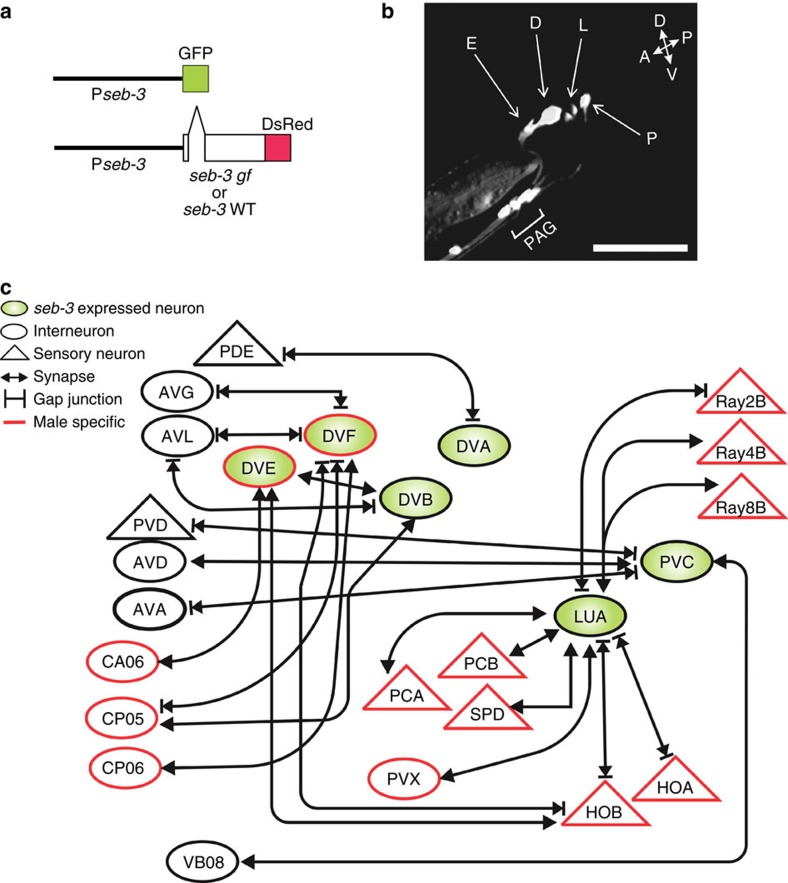
Expression of *seb-3* in the male tail. (**a**) A 3-kb promoter was fused with GFP. The same promoter region drives [SEB-3(gf)::DsRed] in WT and [SEB-3(WT)::DsRed] in *seb-3 lf* mutant. (**b**) Florescent image of a L4 male tail expressing Pseb-3:GFP. D, DVA and DVB; E, DVE and DVF neurons; L, LUAL and LUAR; P, PVC; PAG, pre-anal ganglia. A, anterior; D, dorsal; P, posterior; V, ventral. Scale bar, 10 μM. (**c**) *seb-3*-expressing interneurons and their reciprocal connections in the male tail.

We asked which SEB-3-expressing cell is required to modulate the male's mating tenacity. In ganglia common to both sexes, SEB-3 is expressed in head, ventral cord and tail neurons^18^. Male copulation is controlled by sex-specific circuits in the tail^20^,^22^,^24^,^29^,^32^. We constructed transgenic animals that express a green fluorescent protein (GFP) reporter fused to 3 kb of the *seb-3* upstream promoter region, and found in the male tail, *seb-3* is expressed in combinations of sensory neurons, motor neurons and interneuron that have reciprocal connections^33^. The gene is expressed in DVA, LUA, PVC, DVE, DVF, preanal ganglion motor neurons, in DVB and in one neuron of ray 1 (Fig. 5a–c).

**Figure 6:**
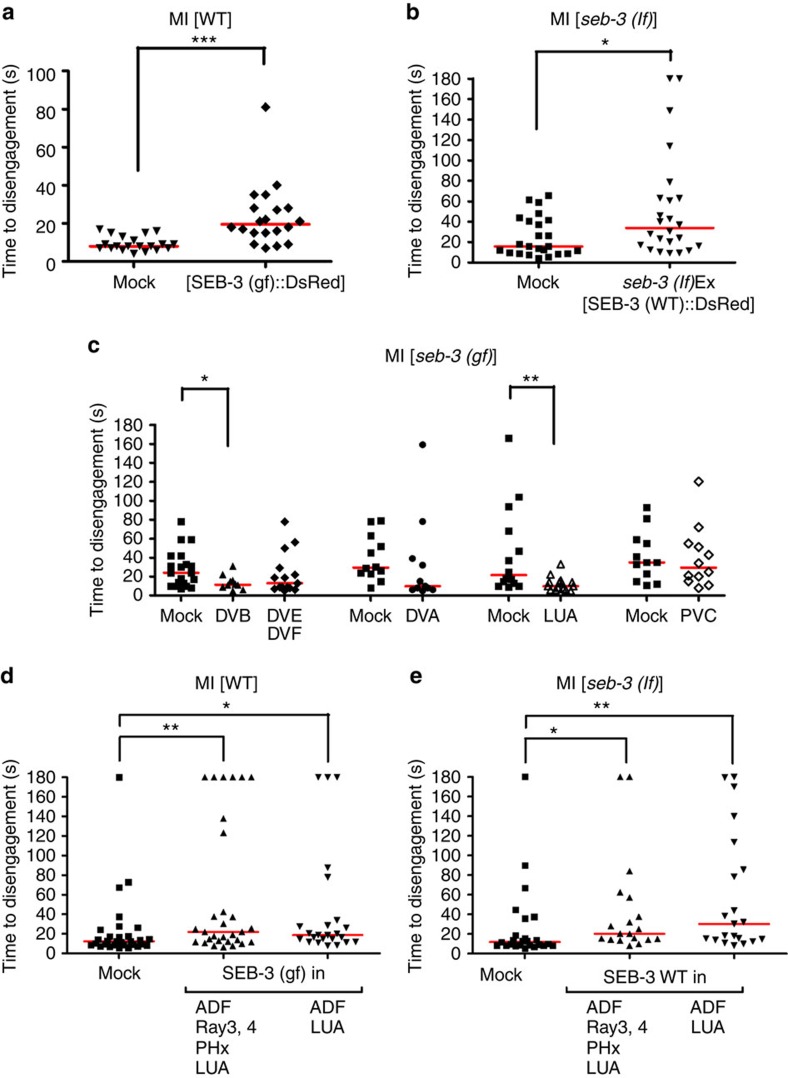
Potentiation of mating by SEB-3 drive requires LUA neurons. (**a**) Measurement of transgenic animals' time to disengagement (*y* axis) in response to 500 μW mm^−2^ blue light exposure (*N*=20 and 21). (**b**) Measurement of transgenic animals' time to disengagement (*y* axis) in response to 450 μW mm^−2^ blue light exposure (*N*=24 and 24). (**c**) Measurement of time to disengagement when neurons are ablated in a *seb-3(gf)* mutant background. The MI assay was conducted under moderate intensity of blue light (370 μW mm^−2^). *N*=20, 10, 15, 12, 12, 14, 16, 12 and 12 (from left to right in order of each group). (**d**,**e**) Neuron-specific potentiation of the male mating circuit. (**d**) SEB-3(gf) was expressed in the indicated neurons (*x* axis) of wild-type males. *N* number is 30, 30 and 24 (from left to right in order of each group). (**e**) SEB-3(WT) was expressed in the indicated neurons (*x* axis) of *seb-3(lf)* males. *N*=25, 19 and 21 (from left to right in order of each group). The MI assay was conducted under 400 μW mm^−2^ blue light (**d**,**e**). *P<0.05, **P<0.01 and ***P<0.001 (Mann–Whitney test).

We confirmed the *seb-3*-expressing neurons were sufficient to potentiate mating behaviour. When WT males expressed the functional *seb-3(eg696gf)*::DsRed fusion complementary DNA from the 3-kb promoter region (Fig. 5a), they displayed enhanced mating drive characteristics. The copulating transgenic males showed a higher tolerance to blue light, similar to the genetic *seb-3(gf)* mutant (Fig. 6a). Similarly, *seb-3(lf)* mutant males were also rescued by the SEB-3(WT)::DsRed receptor (Fig. 6b).

We next asked whether tail neuron(s) were required for *seb-3* to potentiate mating behaviour. We laser-ablated the *seb-3*-expressing cells in the *seb-3(gf)* mutant male tail and tested whether their enhanced mating drive was suppressed in the MI assay. Of the six posteriorly located *seb-3*-expressing neurons, the DVA and DVB have the fewest connections to neurons involved in male mating behaviour (Fig. 5c). DVA ablation has little effect on the time to disengagement; however, DVB-ablated *seb-3(gf)* males show decreased tenacity to mate in the Mi paradigm (Fig. 6c) and required a longer time to initiate mating (Supplementary Fig. 2a). This defect may not be related to *seb-3*, as DVB-ablated worms display additional defects; the posterior intestinal cells are mis-positioned and press on the vas deferens region of the gonad (Supplementary Fig. 2b,c). As the somatic gonad is required to promote mating behaviour, disrupting its function in this manner causes the males to display gonad-ablated characteristics^29^. The mispositioned posterior intestinal cells could be due to abnormal intestinal muscle contractions, caused by the lack of DVB-induced GABA neurotransmission^34^.

Of the remaining four *seb-3*-expressing neurons, the LUA makes reciprocal connections with the multi-functional PCA, PCB, HOA and HOB sensory neurons, which redundantly act with the rays to promote backward locomotion, sense features of the vulva and initiate spicule insertion attempts (Fig. 5c)^20^,^33^. We found that the lack of LUA reduced the enhanced mating drive of *seb-3(gf)* males, whereas removing DVE and DVF or PVC did not cause a significant effect in the MI assay (Fig. 6c). Similar to earlier experiments, we confirmed that ablating LUA did not change the male's ability to sense blue light (Supplementary Fig. 3) but reduced its ability to sustain copulation in the presence of the irritant. LUA is the most interconnected of the *seb-3*-expressing neurons and these results are consistent with the possibility that reciprocal connections between LUA and male-specific circuits might potentiate copulation behaviour (Fig. 5c)^33^.

The cell ablation experiments identify LUA as an important neuron for SEB-3 to potentiate mating behaviour, but they do not specify whether SEB-3 functions in LUA or whether this neuron is part of the circuit required for SEB-3 to potentiate mating. To address this issue, the *Neurospora* repressible Q binary expression system, adapted to *C. elegans* by Wei *et al.*^35^, was used to limit SEB-3 expression to specific neurons. We then asked whether the limited expression of SEB-3 was capable of potentiating male mating*. pgpa-10*:QF combined with *ptrx-1*:QS limited *seb-3(eg696gf)* expression in WT males and *seb-3(WT)* expression in *seb-3(lf)* males, to a sensory neuron pair in the head (ADF), a pair of ray neurons in Ray 3 and Ray 4, a pair of phasmid sensory neurons (PHx) and the LUA interneurons in the tail. In parallel, we also used *klp-6*:QS to further limit expression to ADF and LUA. In both cases, we observed that the copulation behaviours of WT (Fig. 6d) and *seb-3(lf)* (Fig. 6e) transgenic worms persisted longer in the presence of the blue light irritant. We do not believe that SEB-3 or SEB-3(gf) expressed in the ADF neurons are the reason that the transgenic males show mating persistence in the irritant, as the native *seb-3* promoter does not express the receptor in these sensory neurons^18^. Thus, taken together, the transgenic expression and laser ablation experiments suggest that the LUA interneurons play a role in SEB-3 reinforcement of sexual drive.

### LUA activity increases during different steps of copulation

**Figure 7:**
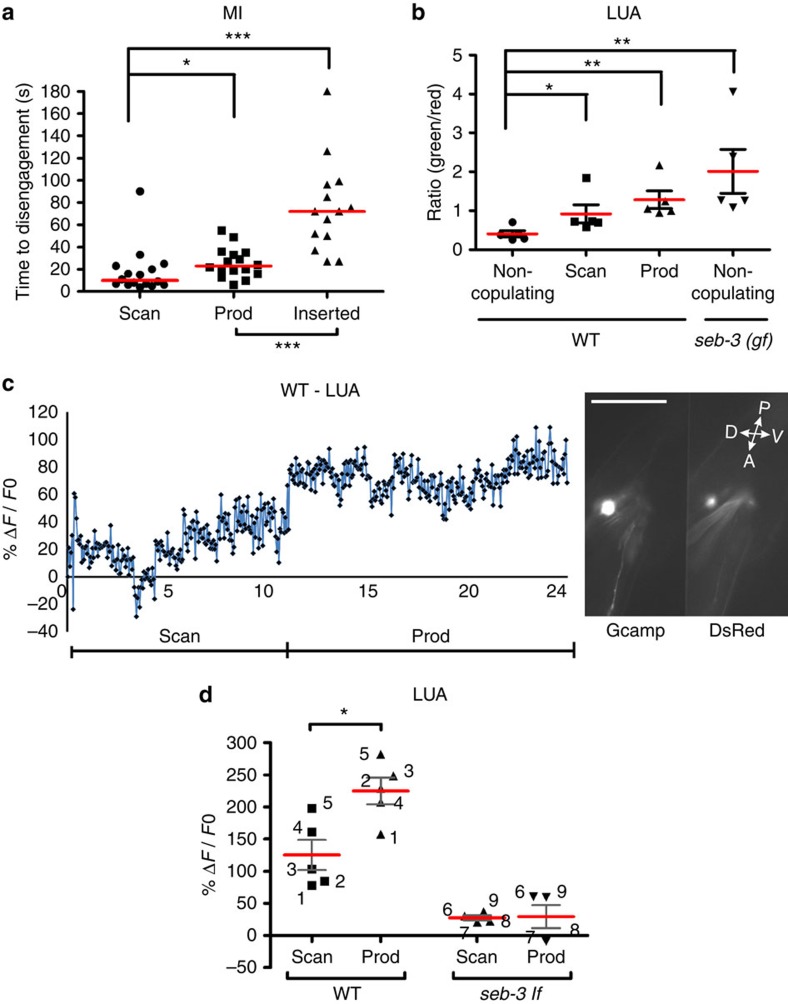
SEB-3 potentiates mating drive via the LUA neurons. (**a**) The MI assay (50 μW mm^−2^) was conducted for each step of copulation with vulva-containing hermaphrodites (*x* axis). Bar represents median as shown in other MI assay and *N*=16, 16 and 14 (from left to right in order of each group). (**b**) Ca^2+^ transients in LUA neurons of non-copulating and copulating males. The *y* axis represents the ratio of G-CaMP (green) to DsRed^61^ fluorescence during the indicated behaviours. Bar represents mean with s.e.m. and *N*=5 for each group. (**c**) Ca^2+^ transients in LUA neurons of one male during scanning and prodding mating behaviours. The *x* axis is time in seconds. The *y* axis is %Δ*F*/*F*_0_. The image is of the LUA fluorescence in a 1-day-old adult male. A, anterior; D, dorsal; P, posterior; V, ventral. Scale bar, 10 μM. (**d**) Summary of Ca^2+^ transients of five males analysed as in **c**. Each point is the average of the %Δ*F*/*F*_0_ during the indicated behaviour (*x* axis). Each number represents an individual male. Bar represents mean with s.e.m. and *N*=5 (WT) and 4 (*seb-3lf*). *P<0.05 and ***P<0.001 (Mann–Whitney test). Inserted, male fully inserting his spicules into the vulva; Prod, male prodding its copulatory spicules at the vulva slit; Scan, male scanning the hermaphrodite cuticle.

As we suggest that SEB-3 is acting in LUA to increase the male's tolerance to 475-nm (blue) light, it is possible that LUA activity changes during mating to modulate this behaviour. To address this, we first established whether changes in the male's tolerance to 475-nm (blue) light are correlated with different steps in mating. We hypothesized that if the male's sexual drive increases as he approaches consummation (ejaculation), then so will its tolerance to blue light. Using static vulva-containing hermaphrodites as a mating partner, blue light was applied at different steps of copulation: backward scanning, spicule intromission attempts and ejaculation (Fig. 7a). We observed that during spicule intromission attempts, the male's tolerance to blue light was higher than when it was scanning. Throughout sperm transfer, the male displayed even higher tolerance to the blue light irritant. However, during and after ejaculation, the male is lethargic^29^, which might mask responses to the applied stimulus.

**Figure 8:**
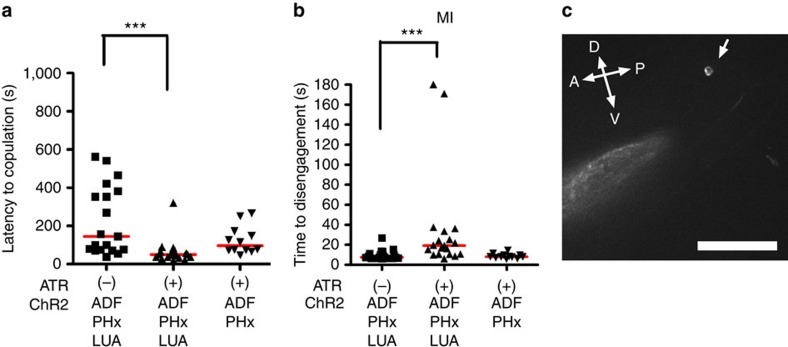
Optogenetic activation of LUA is sufficient to enhance mating drive and tenacity of copulation. ChR2::YFP is expressed in the ADF, PHx and LUA of *seb-3 (lf)* males using the Q binary expression system. (**a**) Latency to initiate copulation before blue light exposure. *N*=19, 19 and 12 (from left to right in order of each group). ‘ATR' refers to all*-trans* retinal. (**b**) The MI assay was conducted under 450 μW mm^−2^ intensity of blue light in [ChR2::YFP]-expressed *seb-3 (lf)* males. *N*=19, 19 and 12 (from left to right in order of each group). ‘ATR' refers to all*-trans* retinal. (**c**) Arrow indicates LUA neurons that express [ChR2::YFP] in the L4 male tail. Scale bar, 10 μM. ***P<0.001 (Mann–Whitney test).

As males that were engaged in spicule intromission attempts displayed higher tolerance to blue light, we asked whether LUA activity also increases during this step. Spicule intromission behaviour is initiated by the PCA, PCB and PCC postcloacal sensory-motor neurons. Previous work has shown that when the male's cloaca contacts the vulval lips, the activity of these neurons increases^29^. As the LUA interneurons are reciprocally connected to PCA and PCB (Fig. 5c), we asked whether the LUA activity also increases on spicule intromission attempts. We monitored WT LUA Ca^2+^ transients using the Ca^2+^ sensor G-CaMP in copulating *lite-1*-deficient males and compared the fluorescence changes against the steady fluorescence of co-expressed DsRed^36^,^37^,^38^. We used the *lite-1* mutation, as 475-nm (blue) light is the wavelength used to excite the G-CaMP fluorescence. As expected, LUA Ca^2+^ transients were higher in mating males compared with non-mating males (Fig. 7b). In addition, consistent with increased blue light tolerance, LUA activity was higher during spicule intromission attempts than when the males were scanning for the vulva (Fig. 7b). When we analysed the transition between scanning and intromission (Fig. 7c), we determined that the LUAs' Ca^2+^ transients increased ∼80% between these mating steps in WT, but not in *seb-3 (lf)* males (Fig. 7d). The LUA Ca^2+^ levels were also measured in *seb-3(gf)* males, but the LUA G-CaMP fluorescence in non-copulating mutant males was already much higher than in the WT (Fig. 7b). The high starting fluorescence precluded our ability to measure further increases. We then asked whether activation of LUA, independent of SEB-3, can promote copulation in blue light. In *seb-3 (lf)* males, we activated LUA using the channelrhodopsin2 (ChR2) ion channel. To prime the male's sex drive, ChR2 males were pre-stimulated with 475-nm light 5 min before copulation. We then measured how fast they initiated copulation and how long they endure blue light^39^. We found that ChR2 activation in the LUA and PHx caused males to initiate copulation faster and also display increased mating tenacity. In contrast, transgenic mosaic males that expressed ChR2 only in the PHx neurons did not display these behaviours, suggesting that activated LUA is sufficient to increase mating drive (Fig. 8a,b). Taken together, our data indicates that increases in LUA activity correlate with the male's tenacity to copulate in the presence of blue light stress; however, this raises the question of whether SEB-3 modulates male's sexual drive in other stress conditions.

### Acclimation to different stresses uses SEB-3 in LUA

**Figure 9:**
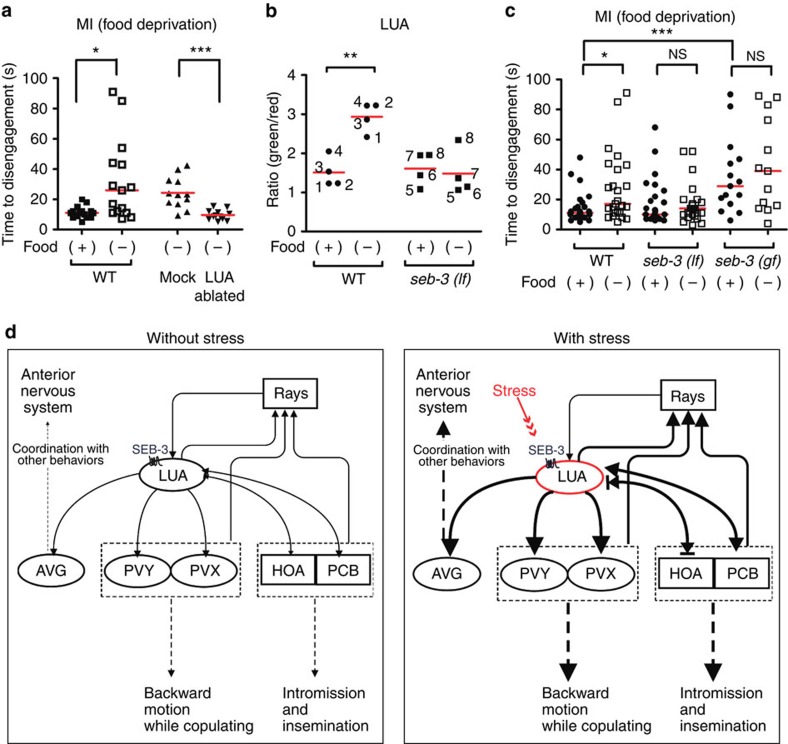
Stress induces enhanced mating drive through activation of the SEB-3. (**a**) The MI assay (450 μW mm^−2^) was conducted on WT males in well-fed and food-deprived conditions and on LUA neurons-ablated WT males in the food-deprived condition. *N*=15, 15, 12 and 12 (from left to right in order of each group). (**b**) Ca^2+^ transients in LUA neurons of non-copulating WT males and *seb-3 (lf)* males. The *y* axis represents the ratio of G-CaMP (green) to DsRed^61^ fluorescence with or without food for 120 min (*x* axis). Each number indicates the same individual male in the presence and absence of food. *N*=4 for each group. (**c**) *seb-3* mutant respond to food deprivation during MI assay conducted using 450 μW mm^−2^ blue light. *N*=29, 25, 25, 25, 15 and 13 (from left to right in order of each group). *P<0.05, **P<0.01 and ***P<0.001 (Mann–Whitney test). (**d**) Model of stress-elicited enhanced mating drive via LUA in mating circuits.

Previous work implicated food stress in modulating male mating^23^,^24^. In particular, food deprivation can enhance the sexual potency of aged males and suppress genetic mutant phenotypes that disrupt copulation^38^,^40^. Therefore, we asked whether food deprivation has an impact on the male's response to 475-nm (blue) light during mating using the MI assay. When WT males are acclimated to food deprivation (Supplementary Figs 4–9), latency to copulate was still much longer and more variable than in well-fed males. Food-deprived WT males displayed 434.1 s of latency to copulate (s.d.±346.2, *P*=0.0008 by Mann–Whitney test), whereas well-fed males showed 164.4 s (s.d.±100.4). However, when the stress-acclimated males did copulate, they displayed increased tolerance to 475-nm (blue) light (Fig. 9a). Thus, food-deprived males are less responsive to the additional application of noxious stimuli.

This reduced response to blue light stress under food-deprived conditions could be due to an increase in LUA activity, as we have already shown that increased LUA activity correlates with decreased response to noxious 475-nm (blue) light (Fig. 7b). Therefore, we asked whether LUA ablation compromised copulation behaviour under stress. LUA-ablated males showed impaired food deprivation-induced enhanced tenacity of copulation (Fig. 9a). We also asked whether food deprivation increases LUA activity and found that Ca^2+^ transients increase in non-copulating WT males (Fig. 9b). Thus, these neurons might potentiate the male mating circuit to promote continued copulation in the presence of multiple adverse stimuli.

As we have shown that *seb-3* works in the LUA neurons to mediate the response to acute light stress in males, we asked whether increased *seb-3* function is required for males to copulate in food stress conditions. In addition, *seb-3* has been shown to regulate how hermaphrodites respond to general stress, increasing the likelihood it is involved during food deprivation^18^. We measured LUA activity in non-copulating food-deprived *seb-3 (lf)* males and found that LUA Ca^2+^ transients were not increased on starvation (Fig. 9b). It is consistent with the idea SEB-3 can promote LUA activity under food stress. We also assayed how fed and starved *seb-3(lf)* and *seb-3(gf)* males behaved in the MI assay. Unlike WT, the starvation treatment did not change how both mutants respond to blue light (Fig. 9c). Regardless of the feeding state, *seb-3(gf)* males were more tolerant to blue light than fed or starved *seb-3(lf)* males. In addition, *seb-3(gf)* males displayed similar blue light tolerance to starved WT males. Thus, males lacking a modifiable SEB-3 are unable to respond to nutrient deprivation. Taken together, we propose that in WT males, stress conditions may modulate SEB-3 activity in the LUA neurons, so that the interneurons can potentiate signals in the interconnected copulatory circuits (Fig. 9d)^33^.

## Discussion

In this work, we explored how the persistence of a motivated behaviour is modulated. Our results show that the SEB-3 GPCR can potentiate neural circuits involved in copulation so that the male *C. elegans* can sustain copulation attempts under mild stress conditions such as noxious light and food deprivation.

SEB-3 is a member of the secretin/B family of GPCRs. In higher animals, the B family of GPCRs have been implicated in modulating various physiological responses such as nociception, circadian rhythms, ion transport, osmoregulation, nutrient transport and stress response^41^. *C. elegans* encodes five secretin/B-like GPCRs genes: *seb-2*, *lat-1*, *lat-2*, *pdfr-1* and *seb-3* (ref. 42). *seb-2* has not yet been characterized. *lat-1* and *lat-2* are homologues of the mammalian latrophilin receptor and LAT-1 has been shown to promote embryonic developmental elongation in *C. elegans*^43^. In contrast, PDFR-1 is related to the *drosophila* pigment dispersing factor GPCR and also calcitonin GPCR. This receptor acts in gender-common neurons to promote locomotor and reproductive behavioural arousal^25^,^44^. Similar to PDFR-1, SEB-3 is also involved in regulating the intensity of arousal. SEB-3 is most related to the CRF receptor family, having 28–30% sequence identity and 46–48% similarity to the mammalian Gαs-coupled stress-related GPCRs^18^.

The mammalian CRF GPCRs have been studied in the anterior pituitary. The receptor is activated by CRF from the hypothalamic paraventricular nucleus. The anterior pituitary then secretes adrenocortotropin hormone to the adrenal cortex. Together, these three brain regions initiate whole animal physiological responses to stress. In addition to the HPA axis, the mammalian CRF receptors are also expressed in the extended amygdala, where they regulate the proper behavioural and psychological reactions to external stress. In humans, addiction, anxiety, depression and other psychological disorders are linked to dysregulation of CRF signalling in the extended amygdala. Consistent with this, studies using mouse genetic knockout mutants suggest that activated CRF1 receptor promotes stress reactions (flight or freeze responses), whereas the CRF2 receptor dampens or manages rodent stress^45^,^46^,^47^,^48^. CRF1 receptor antagonists that can reduce stress-induced HPA axis activation in rodents have been identified; however, their efficacy in human clinical trials have been inconsistent^49^,^50^,^51^. In contrast to a strict dualistic view on CRF1 and CRF2 receptor function, a current idea is that in their specific brain regions, the CRF receptors act together so that the animal produces a proper stress response proportional to the intensity of stress^52^. In our study, we add to this idea by suggesting that SEB-3 acts in specific cells to modulate the animal's motivational state, which in turn also affects how the organism responds to stress.

In contrast to the sequence homology between SEB-3 and the mammalian CRF receptor's transmembrane and cytosolic loop regions^18^, none of the 250 annotated *C. elegans* neuroactive peptides show clear sequence homology with mammalian CRF or urocortin peptides^53^. Possibly, this is because the extracellular ligand-binding region of SEB-3 has diverged from the CRF receptor^18^. Several *C. elegans* neuropeptides were identified as ligands of mammalian GPCR orthologues using biochemical approaches. For example, pigment dispersing factor (PDF)-like peptides in *C. elegans* were discovered by peptidomic identification^54^ and pharmacological screening revealed ligands for the *C. elegans* NPY receptor homologue^55^,^56^. Similar biochemical screening methods need to be used to identify SEB-3's ligands.

Male copulation in *C. elegans* is a motivated behaviour regulated by sex-specific circuits^23^,^24^,^25^. We find that SEB-3 modulates male sexual arousal by upregulating the activity of the sex-common LUA interneurons. LUA are bilateral glutamatergic interneurons in the tail^57^. In hermaphrodites, they make synapses with command interneurons that regulate the direction of locomotion^58^. Based on their connectivity with other circuits, the LUA has been suggested to act as a conduit between sensory touch receptors and the locomotion circuit; however, laser ablation of the LUA does not affect how animals respond to acute mechanosensation^59^. In contrast to the hermaphrodite, the male LUA interneurons are wired to multiple male-specific neurons used for copulation behaviour^33^.

Male mating is guided by sensory neurons located in sexual structures in the tail. These sensory neurons connect with additional interneurons and motor neurons to control 64 sex-specific muscles and the gonad^60^. LUA makes presynaptic connections with many of these male copulation-specific neurons, with some of the connections also being reciprocal (Fig. 5c)^33^. Mating behaviour initiates when the tips of the male's 18 bilateral pairs of sensory rays brush against the hermaphrodite's cuticle. If the male's mating drive is low, it will continue on its current locomotor trajectory^29^. However, if the mating drive is high, it will press its body against the hermaphrodite's cuticle and continue to move along its body backwards, scanning for the vulva^61^. The backwards scanning behaviour not only uses the sensory ray neurons but also the putative mechanosensory postcloacal sensillae neurons (PCA, PCB and PCC), the chemosensory hook neurons (HOA and HOB)^20^ and the PVY and PVX interneurons^62^. From the male wiring diagram and the data presented in this work, we suggest that activated SEB-3 (either through its natural ligand interaction or through the *seb-3(gf)* mutation) induces LUA to potentiate its postsynaptic partners PCB, HOA, PVY, PVX and the sensory ray neurons. This could allow a limited exposure of hermaphrodite chemical or mechanical cues to trigger backwards scanning behaviour. The postsynaptic connections from the ray neurons and the reciprocal connections with HOA and PCB could then amplify LUA's activity, thus further reinforcing copulation behaviour (Fig. 8e). If SEB-3 is not activated, then LUA's postsynaptic partners are not potentiated and thus more or repeated stimulation from the hermaphrodite is needed to trigger copulation. Our findings indicate that after SEB-3 promotes the copulation sequence, the male is also less responsive to extraneous signals, such as aversive stimuli.

In our study, we use the aversion of *C. elegans* to noxious 475 nm (blue) light as an efficient way to assay the male's intensity to copulate. When exposed to blue light at 218 μW mm^−2^ or greater, the *C. elegans* hermaphrodite stops its feeding behaviour, moves forward and increases its locomotion rate, to escape the illuminated area. These responses are mediated by the LITE-1 gustatory receptor^26^,^27^. LITE-1 is expressed in multiple neurons: the M1, M4, M5 and MI in the pharynx; ASK, ADL, ASI, ASH, AVG, AVB, RIM and ADF in the head; and PHA, PHB and PVT in the tail. The receptor (along with its paralogue GUR-3) has been shown to mediate aversive behavioural responses to light-generated hydrogen peroxide^30^. Similar to the hermaphrodite, a free-moving WT male will move forward or change direction to escape the illumination of 450 μW mm^−2^ blue light. However, when copulating, the male will tolerate blue light and attempt to sustain mating in the presence of the irritant. The tenacity to execute copulation in blue light is correlated with the intensity of the male's sex drive (Fig. 4) and is mediated by LUA and SEB-3 (Fig. 6). A response to light elicits a forward locomotion behaviour; forward locomotion is promoted by the gender-common AVB command interneuron^63^. In contrast, when the ray neurons contact the hermaphrodite, they activate the male-specific PVY and PVX interneurons, which then activates the gender-common AVA command interneuron. The AVA promotes copulatory backwards scanning locomotion^62^. As the LITE-1 receptor is expressed in the AVB command interneurons, the neurocircuitry of a copulating male exposed to blue light should be receiving two antagonistic locomotor signals. We hypothesize that both the escape circuitry and the copulation circuitry are co-functioning. However, depending on the male's behavioural state, if the activity of SEB-3 is stronger than the activity of LITE-1, then the compulsion of the male to mate will supersede its aversive light response.

Our results also reveal that SEB-3 is not only involved in modulating behaviour under acute light irritation, but functions for acclimation to chronic low-intensity stress, such as food availability fluctuations (Fig. 9a–c). Similar to hermaphrodites, a roaming male subjected to food stress alters its locomotor behaviour, presumably to escape the stress conditions. However, if the male acclimates to the imposed condition and re-establishes its drive to mate, it displays greater endurance to acute blue light. We do not yet know the ligand for SEB-3 or the presynaptic cells that produce it, but we hypothesize that chronic exposure to the stressful condition stimulates ligand secretion. The SEB-3 ligand should then stimulate the LUA interneurons so that it can potentiate the copulation circuitry and re-establish the male's sexual arousal. This scenario is not unreasonable, as others have suggested that for populations to evolve fitness traits to better thrive under stressful conditions, organisms must first be able to reproduce under stress^64^,^65^,^66^.

## Methods

### Strains

All strains were maintained on nematode growth media (NGM) plates with *Escherichia coli* (OP50). *him-5(e1490)* males were used as WT animals. *let-23(sy1); unc-64(e246); lite-1(ce314)* hermaphrodites were used for vulvaless static mates in the MI assay^67^, otherwise *unc-64(e246); lite-1(ce314)* hermaphrodites were used for the MI assay. *seb-3(eg696)* was used for the *seb-3(gf)* allele and *seb-3(tm1848)* for the *(lf)* allele^18^.

Transgenic strains include the following: him-5(e1490) rgEx658 [Pofm-1:gfp]. him-5(e1490) rgEx659 [(pCJ67 Pseb-3:seb-3(eg696)::mDsRED); Pofm-1:gfp]. pha-1(e2123);him-5(e1490) rgEx680 [(pCJ90 Pseb-3:gfp); pha-1(+)]. pha-1(e2123);him-5(e1490);lite-1(ce314) rgEx431 [Phsp-16:egl-2(n693gf)cDNA; Punc-103E:mDsRed; pha-1(+)]. pha-1(e2123);him-5(e1490);lite-1(ce314) rgEx632 [(pCJ87 Pgpa-10:G-CaMP6::SL2:::mDsRed); pha-1(+)]. pha-1(e2123); him-5(e1490); ite-1(ce314) seb-3(eg696) rgEx632 [(pCJ87 Pgpa-10:G-CaMP6::SL2:::mDsRed); pha-1(+)]. pha-1(e2123);him-5(e1490);lite-1(ce314) rgEx [(pCJ97 Pseb-3:G-CaMP6::SL2:::mDsRed); pha-1(+)]. him-5(e1490);seb-3(tm1848lf) rgEx658 [Pofm-1:gfp]. him-5(e1490);seb-3(tm1848lf) rgEx743 [(pCJ141 QUAS: seb-3(WT)::gfp); (pCJ128 Pgpa-10:QF); (pCJ129 ptrx-1:QS); Pofm-1:gfp]. him-5(e1490);seb-3(tm1848lf) rgEx744 [(pCJ141 QUAS: seb-3(WT)::gfp); (pCJ128 Pgpa-10:QF); (pCJ129 Ptrx-1:QS); (pCJ152 Pklp-6:QS); Pofm-1:gfp]. him-5(e1490) rgEx745 [(pCJ143 QUAS:seb-3(eg696gf)::gfp); pCJ128(Pgpa-10:QF); pCJ129(Ptrx-1:QS); Pofm-1:gfp]. him-5(e1490) rgEx746 [(pCJ143 (QUAS:seb-3(eg696gf)::gfp); (pCJ128 Pgpa-10:QF); (pCJ129 Ptrx-1:QS); (pCJ152 Pklp-6:QS); Pofm-1:gfp]. pha-1(e2123); him-5(e1490); lite-1(ce314) seb-3(tm1848lf) rgEx632 [(pCJ87 Pgpa-10:G-CaMP6::SL2:::mDsRed); pha-1(+)]. him-5(e1490); seb-3 (tm1848lf) rgEx785 [(pCJ173 (QUAS:ChR2::yfp); (pCJ128 Pgpa-10:QF); (pCJ129 Ptrx-1:QS); (pCJ152 Pklp-6:QS); Pofm-1:gfp].

### Behavioural assays

To assess mating behaviour, one 1-day-old virgin WT male was incubated with one 1-day-old vulvaless paralysed hermaphrodite (*let-23 (sy1);unc-64(e246);lite-1(ce314)*) on NGM plates containing a 5-mm *E. coli* (OP50) lawn. All mating assays were recorded with a Hamamatsu CA742–95 digital camera, to determine the response time. The male's activity was digitally recorded for 100 min.

The phototaxis assay was conducted as described in Ward *et al.*^27^. A single virgin male was transferred to an NGM plate covered with fresh OP50. An EXFO X-CITE TM120 light source, equipped with variable illumination intensity control, was used to deliver full spectrum light to a Zeiss Stemi SV11 microscope coupled with a 475-ex filter. Light intensity was determined using a blue-light safety detector (PMA2121) and radiometer (PMA2200) (SOLAR Light Inc., USA). For non-copulating males, blue light was applied 30 s after their transfer to the assay plate; the males were exposed to the light irritant, while they were moving forward. After light exposure, stops and directional changes such as reversals, steep body bends and omega turns were counted as aversive blue light responses. After transfer to a new plate, the mean time to the first direction change without the light irritant was determined as a control (WT, 128 s (s.d.±62.26); *seb-3 (gf)*, 80.82 s (s.d.±32.18); and *seb-3 (lf)*, 93.45 s (s.d.±37.63). Males that copulate with vulvaless hermaphrodites sustain continuous backward movement. When blue light was applied to the copulating male, the first directional change, either a stop or forward movement, was counted as a response to the light irritant.

All males used in our MI assays were 1-day-old adults. A single male was transferred to an NGM assay plate, which contained a 5-mm diameter lawn of OP50 and three 1-day-old *let-23(sy1);unc-64(e246); lite-1(ce314)* hermaphrodites. The latency to copulation was determined from the time the male was transferred to the plate, to when it started backwards locomotion behaviour. One minute after the male started copulation, blue light (475 nm) was applied and the males' responses were digitally recorded. The recordings were stopped when the male disengaged from the hermaphrodite. Under normal conditions, the minimum time that a non-irradiated WT male leaves the hermaphrodites was 180 s; therefore, each MI assay ended after 180 s. Each worm was tested once.

To assay the behaviour of postcoital males, one male was incubated with one *unc-64(e246);lite-1(ce314)* hermaphrodite for 120 min. The male was either used in the MI assay immediately or isolated for an additional 120 or 210 min before being subjected to the MI assay. We later determined whether the hermaphrodite was impregnated by scoring for non-paralysed cross-progeny.

The MI assay was conducted on moderate intensity (370 μW mm^−2^) with cell-ablated *seb-3(gf)* males (to test whether they behaved more like WT), whereas higher intensities (450 and 500 μW mm^−2^) were used for stress-treated WT males and [*pseb-3*:*seb-3 eg696gf*] transgenic males (to test whether they behaved more similar to *seb-3(gf)* mutants). Owing to the *seb-3* (*tm1848lf*) male's range of response at the moderate blue light intensity, the MI assay was conducted at 400 μW mm^−2^ intensity with [*seb-3* (WT)] transgenic males and cell-specific expression of [*seb-3(eg696)gf*]. For acute food stress, the MI assay was performed after 30 min of food deprivation. We used 30 min post-food deprivation as the time to conduct the MI assay, as the subtle reduction in the males' locomotion behaviour suggests that they started to become acclimated to the food-deprivation condition (Supplementary Fig. 4). The assay was conducted on unseeded NGM. A copper ring (A14X18, Fastenal, USA) was used to confine the male with three *let-23(sy1);unc-64(e246);lite-1(ce314)* hermaphrodites. The median value from the distribution of responses was used for central tendency, due to the skewed distribution. Statistical significance was determined by the Mann–Whitney test. *P*-values are denoted as follows: *P<0.05; **P<0.01 and ***P<0.001.

To assess locomotion of males on food deprivation, six young adult males were transferred to unseeded NGM and confined to the recording area using a copper ring (A14X18) and allowed to explore for 120 min without disturbance. Locomotion was recorded at 0, 10, 20, 30 and 120 min after food deprivation and track patterns were analysed using the DIAS software (Sollteck Inc., Oakdale, IA, USA).

### Plasmid construction

Sequences of primers used in this report are found in Supplementary Table 1. The 3-Kb *seb-3* promoter region was PCR amplified from genomic DNA using [Pseb-3F1 and Pseb-3B1] or [Pseb-3F1 and Pseb-3B2] primers and recombined into an ATTP cloning vector, using BP Clonase II (Life Technologies, Grand Island, NY), to generate pCJ8 or pCJ88, respectively. pCJ7 was constructed by inserting the Gateway reading Cassette C.1 (Life Technologies) into the SmaI site of the pPD95.75 Fire vector, which encodes a GFP reporter gene. To make pCJ90 [P*seb-3:gfp*], pCJ88 was recombined with pCJ7 using LR Clonase II (Life Technologies).

The 1.3-Kb *seb-3* cDNA was PCR amplified from the ORFeum library (Open Biosystem) using Infseb-3F1 and Infseb-3B1 primers. The primers InfGW F1 and InfGW B1 were used to introduce a few *seb-3* cDNA sequences in frame with mDsRed into the pGW332DsRed vector. The *seb-3* cDNA PCR product was then recombined with the mDsRed vector using Clontech's Infusion kit (Mountain View, CA) to make pCJ5. PCR errors were corrected and the *eg696(gf)* mutation was introduced by site-directed mutagenesis, using the primers SDM1-F/B, SDM2-F/B, SDM3-F/B, SDM4-F/B and SDM5-eg696-F/B, to make pCJ61 [*seb-3(eg696gf)*::mDsRed]; the location of the *eg696gf* mutation is shown in Supplementary Fig. 10. pCJ8 (P*seb-3*) was recombined with pCJ61 using LR Clonase II to generate pCJ64 [P*seb-3*:*seb-3*(*eg696gf*)::mDsRed]. We then introduced the first intronic region into pCJ64 using Clontech's Infusion kit. A PCR product containing 306 bp of the first intronic region of *seb-3* (amplified using HybIns-F1 and HybIns-B1 primers) was recombined with linearized pCJ64 (via inverse PCR, and HybVec-F1 and HybVec-B1 primers) using the Clontech Infusion kit, to make pCJ67 [P*seb-3*:*seb-3* intron *(eg696gf)*::mDsRed]. The first intronic region of *seb-3* stabilized the expression of the SEB-3*(eg696gf)*::mDsRed protein. The cDNA of *seb-3*(WT) was also generated in a similar way. The 306-bp PCR product (HybIns-F1+HybIns-B1 primers) of the first intronic region was introduced into pCJ61, to generate pCJ70. Next, the *eg696(gf)* mutation was reverted back to WT (A>G) using site-directed mutagenesis, to generate pCJ122 [*seb-3(WT)*::mDsRed]. pCJ8 (P*seb-3*) was recombined with pCJ122 using LR Clonase II, to generate pCJ124 [P*seb-3*:*seb-3(WT)*::mDsRed].

### Specific expression of *seb-3* alleles and ChR2::YFP

Instead of mDsRed, GFP was translationally fused to *seb-3* cDNA and used for cell-specific expression. To fuse GFP to wild-type *seb-3*, the vector pCJ122 and GFP were PCR-amplified using the primers 134Ins-F1+134Ins-B1 and 134Vec-F1+134Vec-B1, respectively, and used in a Clontech Infusion reaction to make pCJ134 [ccdB:*seb-3* (WT)::*gfp*]. In the same manner, the GFP PCR product from pCJ7 and PCR-linearized pCJ70 were recombined using the Clontech Infusion kit, to generate pCJ136 [ccdB:*seb-3*(*eg696gf*)::*gfp*].

To control the expression of the *seb-3* cDNA in a subset of neurons including LUA, we used the Q binary expression system^35^. A 371-bp 5xQUAS region, containing the binding sites of the transcription activator QF, was PCR amplified using attB-5XQUASF1 and attB-5XQUASB1 primers from the plasmid pXW12 (ref. 35). The PCR product was recombined using BP Clonase II with an ATTP entry vector to create pCJ138. pCJ138 was recombined with pCJ134 using LR Clonase II, to generate pCJ141 [QUAS:*seb-3* (WT)::GFP]. In the same way, pCJ144 [QUAS:*seb-3* (*eg696gf*)::GFP] was made using LR Clonase II, pCJ138 and pCJ136.

The transcriptional activator QF and QS (the repressor of QF) were first cloned separately into the ATTP entry vector pTG38. To make pTG38, the HindIII–ApaI region (containing the Nhe1, Kpn1 sites and the *unc-54* untranslated region) of the Fire vector pPD49.26 was cloned into the HindIII–ApaI region of the vector pBR322. The ATTP region from the Invitrogen Gateway entry vector pDONR221 was then cloned into the upstream SmaI site. To clone the QF and QS transcriptional regulators, an Nhe1 QF-containing fragment from the plasmid pXW17 and an Nhe1–Kpn1 QS fragment from the QS-containing plasmid pXW25 (ref. 35) were first subcloned into the Nhe1 site and the Nhe1–Kpn1site, respectively, of pTG38, creating the vectors pLR326(ATTP:QF) and pLR327(ATTP:QS).

To drive QF in cells of interest, the 3-Kb *gpa-10* promoter region was PCR amplified using attBpgpa-10-F1 and attBpgpa-10-B1 primers. The PCR product was recombined with pLR326 using BP Clonase II, to create pCJ128 [*pgpa-10*:QF]. Previously, *gpa-10* was reported to be expressed exclusively in the LUA neurons of the hermaphrodite tail region. However, in males, p*gpa-10* drove gene expression in additional neurons in the male tail, mainly in Ray 3 and Ray 4 neurons and a pair of phasmids in addition to LUA.

P*gpa-10:*QF drives expression in ADF, ASI and ASJ, which ectopically puts SEB-3 in these head sensory neurons. To restrict QF expression, we co-expressed QS using heterologous promoters that contain overlapping expression with *gpa-10*. To suppress mis-expression of *seb-3(gf)* expression in head neurons involved in aversive behaviours, p*trx-1*, which is reported to drive the expression in ASJ and ASI^68^, was PCR amplified using attB-trx-1F and attB-trx-1B. Using BP Clonase II, the PCR product was recombined with pLR327 to generate pCJ129 [p*trx-1*:QS]. p*klp-6* is reported to drive expression in Rays (RnBs 1–9 but not 6), HOB, IL2 and CEM^69^. p*klp-6* was PCR amplified using attB-klp-6F and attB-klp-6B primers, and pCJ44 [p*klp-6*:CeKR] as a template. The PCR product was then recombined with pLR327 using BP Clonase II, to create pCJ152 [p*klp-6*:QS].

To control the expression of ChR2 in a subset of neurons including LUA, we used the Q binary expression system in the same manner. The construct pCJ138, containing the 371 bp 5x QUAS sequence, was recombined with pLR167 (ref. 29), using LR Clonase II, to generate pCJ173 [QUAS:*ChR2::yfp*]. To express pCJ173 [QUAS:*ChR2::yfp*] in the LUA, pCJ128 [*pgpa-10*:QF], pCJ129 [p*trx-1*:QS] and pCJ152 [p*klp-6*:QS] was co-injected to *pha-1(e2123);him-5 (e1490);seb-3 (tm1848)*.

### Cell-specific expression of G-CaMP6

pLR305 [Gateway Cassette C.1:G-CaMP6::SL2:::mDsRed] was constructed as described in LeBoeuf *et al.*^29^. To drive G-CaMP6 expression in the LUA neurons, P*seb-3* and P*gpa-10* were used^70^. The 3-kb *gpa-10* promoter region was PCR amplified using attBpgpa-10-F1 and attBpgpa-10-B1 primers from [pENTR:*gpa-10*] and recombined with an ATTP entry vector using BP Clonase II, to generate pCJ85. pCJ85 was recombined with pLR305 using LR Clonase II, to make pCJ87 [P*gpa-10*:G-CaMP6::SL2:::mDsRed]. pCJ88 (P*seb-3*) was recombined with pLR305 using LR Clonase II, to make pCJ91[P*seb-3*:G-CaM6:SL2::mDsRed]. Next, we introduced the first intronic region of *seb-3* into G-CaMP6, similar to pCJ67. Two PCR products, using the GCamp6-seb-3Int.F1/B1 primers and HybGCamp6-F1/B1 primers, were recombined using the Clontech Infusion kit, to make pCJ97 [P*seb-3*:G-CaMP6 intron::SL2:::mDsRed].

### Transgenic animals

The concentration of the final injection mixtures were 200 ng μl^−1^. pBX-1, which contains *pha-1(+*), was co-injected at 50 ng μl^−1^ into *pha-1(e2123)* as an injection marker. A P*ofm-1:gfp* plasmid was co-injected at 50 ng μl^−1^ into WT as an injection marker. pCJ90, 87 and 97 were injected at 50 ng μl^−1^. pCJ67 and pCJ124 were injected at 20 ng μl^−1^. pCJ141, pCJ144, pCJ128, pCJ129 and pCJ152 were injected at 30 ng μl^−1^. pCJ173 was injected at 10 ng μl^−1^.

### Cell ablation

Cells were ablated using a Spectra Physics VSL-337ND-S Nitrogen Laser (Mountain View, CA) connected to an Olympus BX51 microscope (Olympus, PA, USA)^60^. Ablations and mock-ablated control animals were conducted at L3 stage on 5% noble agar pads with 5 mM NaN_3_. Animals were kept on the NaN_3_ pads for <5 min.

### Optogenetic activation-induced behaviour

Brightly expressing ChR2::YFP *seb-3(lf)* L4 males were selected and incubated on NGM containing all-*trans* retinal (A.G. Scientific, San Diego, CA) for 17 h. Plates were freshly prepared with 50 μM all-*trans* retinal and OP50 bacteria. We presumed that normally, activated *seb-3* receptors modulate the male's copulatory drive before physical mating contact. To artificially approximate this with ChR2 instead of SEB-3, one male at a time was exposed to 475 nm wavelength light on a plate (± all-*trans* retinal) for 5 min to prime the ChR2-expressing cells. The male was then immediately transferred to an assay (± all-*trans* retinal) plate with three vulvaless static mates. The behaviour was digitally recorded, to determine the latency to initiate copulation and the time to disengagement in the presence of 450 μW mm^−2^ blue light (475 nm). After the MI assay, ChR2::YFP-expressed cells were verified in each male using an epifluorescence-equip Olympus BX51 microscope.

### Ca^2+^ imaging

Ca^2+^ transients were measured using the Ca^2+^ sensor G-CaMP in free-moving and copulating *lite-1(ce314)* males, as described in Correa *et al.*^32^ and LeBoeuf *et al.*^29^. A 1-day-old transgenic virgin male was transferred to a seeded (OP50) or unseeded NGM plate. To compare Ca^2+^ transients in LUA neurons of non-copulating WT males with and without food, males were withdrawn from OP50 for 120 min. One hundred and twenty minutes of food deprivation was used as the assay time, as the reduction in the males' locomotion indicated that they had acclimated to the food stress condition (Supplementary Figs 4–9). Males were manually tracked under a × 10 or × 20 objective of an Olympus BX51 microscope (Olympus). The G-CaMP and mDsRed emission signals were recorded using a Dual View Simultaneous Imaging Systems with an OI-11-EM filter (Photometrics, Surrey, BC, Canada) and Hamamatsu ImagEM Electron multiplier (EM) charge-coupled device camera. The digitally recorded green fluorescence in the moving male was compared with the co-expressed red fluorescence. Data were analysed using SimplePCI 6.0 and Microsoft Excel as described in LeBoeuf *et al.*^29^.

Ten 2-day-old, heat-shocked, paralysed *pha-1(e2123);him-5 (e1490);lite-1(ce314) rgEx431* [P*hsp-16:egl-2(n693gf)cDNA;*P*unc-103E:mDsRed;pha-1(+)*] hermaphrodites were placed on a 5-mm diameter OP50 lawn. A 1-day-old transgenic virgin male was transferred to the lawn and when he initiated copulation, the excitation illumination and digital recording was initiated. G-CaMP and mDsRed signals were analysed for each step of copulation as described above.

### Data availability

The authors declare that the data supporting the findings of this study are available within the article and its Supplementary Information files, or available from the authors upon request.

## Additional information

**How to cite this article:** Jee, C. *et al.* CRF-like receptor SEB-3 in sex-common interneurons potentiates stress handling and reproductive drive in *C. elegans*. *Nat. Commun.* 7:11957 doi: 10.1038/ncomms11957 (2016).

## Supplementary Material

Supplementary Information Supplementary Figures 1-10, Supplementary Table 1
