# Supplementary material for: CRF-like receptor SEB-3 in sex-common interneurons potentiates stress handling and reproductive drive in C. elegans
Source: Nat Commun. 2016 Jun 20;7:11957. doi: 10.1038/ncomms11957 (PMC4915151; doi:10.1038/ncomms11957)
Supplement: Supplementary Information — Supplementary Figures 1-10, Supplementary Table 1 [file ncomms11957-s1.pdf]

Supplementary Figure S1 (related to Fig. 1 and 3)

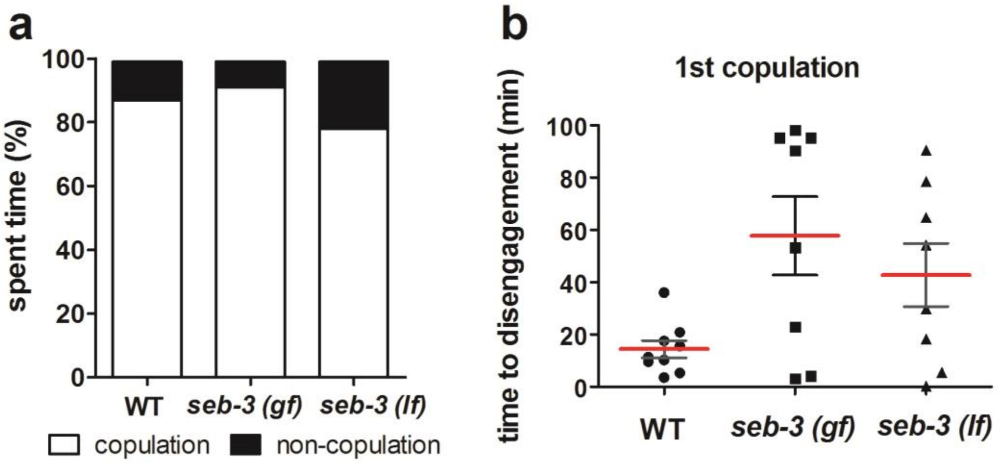

Persistence of male mating with a vulvaless hermaphrodite. A virgin wild-type, *seb-3 (gf)*, and *seb-3 (lf)* male was paired with a vulvaless, non-moving hermaphrodite on a NGM plate with *E. coli* as a food source. (a) Copulatory behavior of a single male with a vulvaless static hermaphrodite was recorded for 100 min. A copulation session was determined as the duration from contact to disengagement. The amount of time spent copulating without extinction was represented by summing each copulation session (n=8). Copulation behavior of both *(gf)* and *(lf)* males with vulvaless static mates were similar to wild-type. However, *seb-3 (gf)* male spent more time to copulate whereas *seb-3 (lf)* males showed the opposite, whose tenacity of copulation is consistent with MI assay results. P=0.0233 by Chi square analysis [χ^2 (2, N=8)=7.527]. (**b**) The Y-axis represents the duration of the first copulatory time. Bar represents mean on SD. Both *seb-3 (gf), and seb-3 (lf)* male exhibited longer time to disengagement on their first copulation with an average time of 57.74 min (SD± 42.43) and 42.75 min (SD± 34.08) respectively, however not significant due to the SD (P= 0.0927 by Mann-Whitney test). N=9 (WT), 8 (*seb-3gf*), and 8 (*seb-3lf*).

Supplementary Figure S2 (related to Fig. 6)

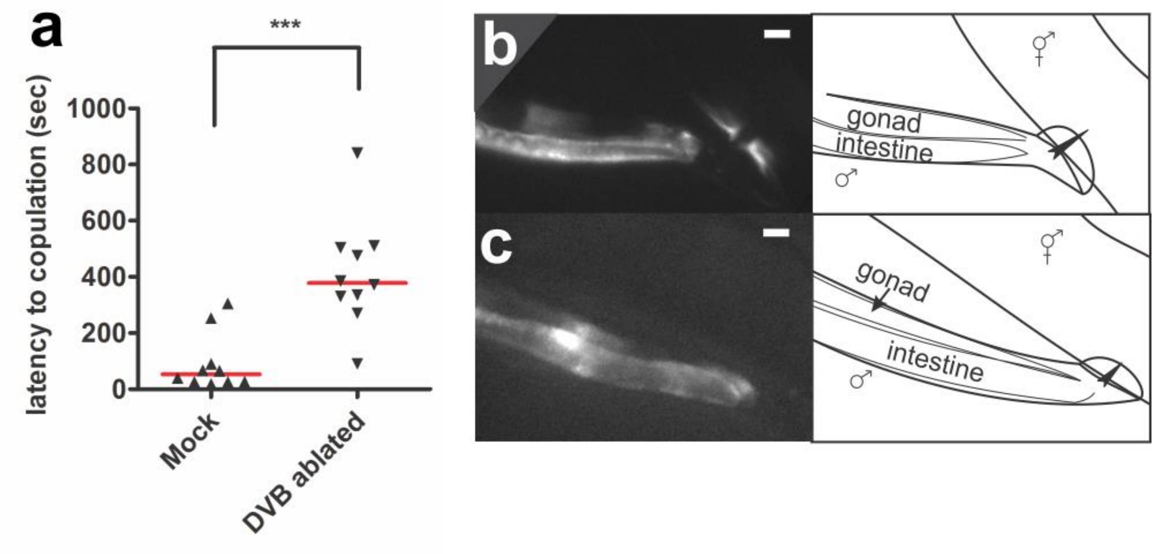

DVB ablated male showed enlarged posterior intestine, resulting in shrunken gonad, and was less interested in copulation.

(a) The latency to initiate copulation before MI approach was shown in DVB ablated male. (b) Mock ablated male showed extended gonad after insertion of spicules, which leads to smooth sperm transfer (***, P <0.001 by Mann-Whitney test). (c) DVB ablated male represented enlarged posterior intestine to block extension of gonad for sperm transfer. N=10 for each group. Scale bar = 10μM.

Supplementary Figure S3 (related to Fig. 6)

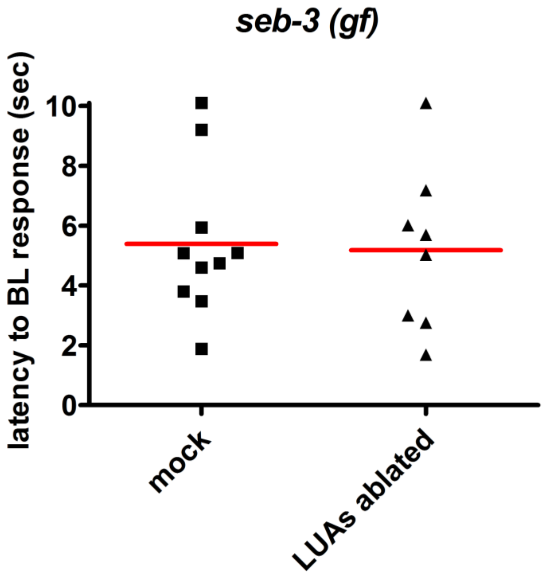

Laser ablation of LUA cells did not change blue light sensitivity of *seb-3 gf* mutant males. A non-copulating male was exposed to blue light (370 mW/mm^2), at the same intensity as that in Fig. 6c. and the latency to respond (to stop movement and change direction) was determined.

Supplementary Figure S4 (related to Fig. 8)

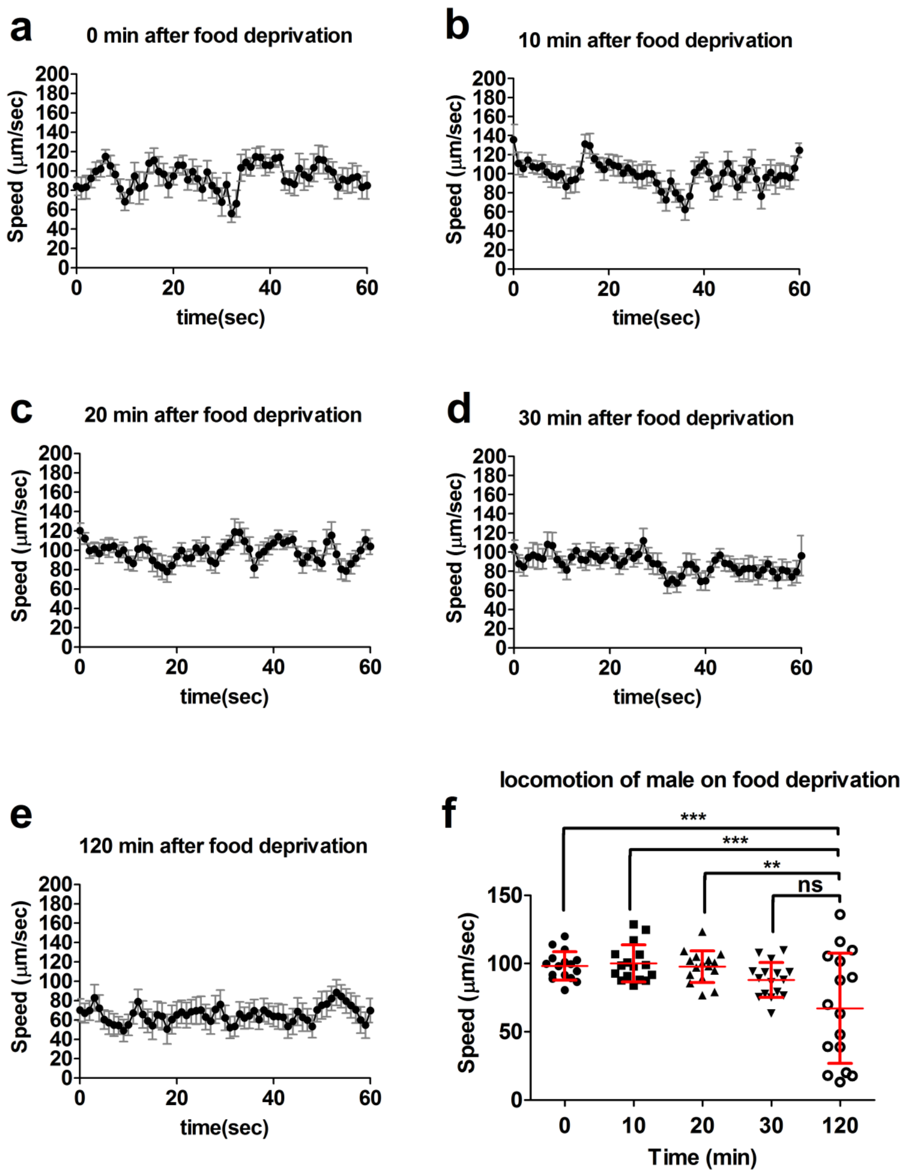

Average of speed (N=16) was displayed at 0 min (a), 10 min (b), 20 min (c), 30 min (d), and 120 min (e) after food withdrawal. (f) represents the speed of each male (N=16) on food deprivation. Wild-type males were acclimatized to food deprivation. Their locomotion was reduced after food deprivation due to increasing their copulation

behavior by themselves. 30 min after starvation, their locomotion was reduced and 120 min later after food deprivation, it decreased dramatically (Statistical significance was determined by One-way ANOVA. $F[4, 75] = 6.774$, $p<0.0001$ and P values are denoted as follows: **, $P<0.01$; ***, $P<0.001$ by Tukey's multiple comparison test). N=16 for each group.

Supplementary Figure S5 (related to Fig. 8)

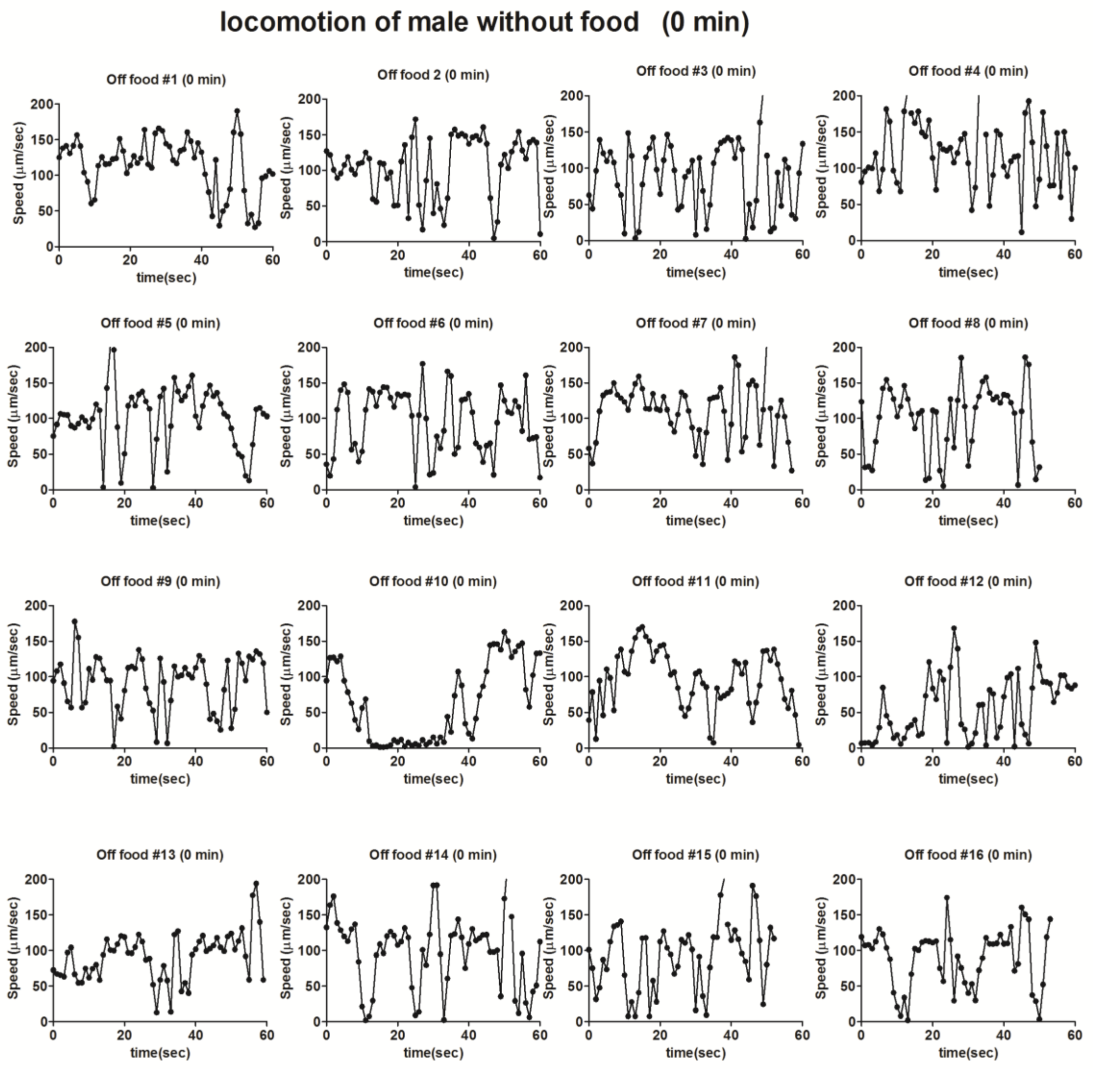

The locomotion of an individual male without food (0 min) shown in Figure S4a.

Supplementary Figure S6 (related to Fig. 8)

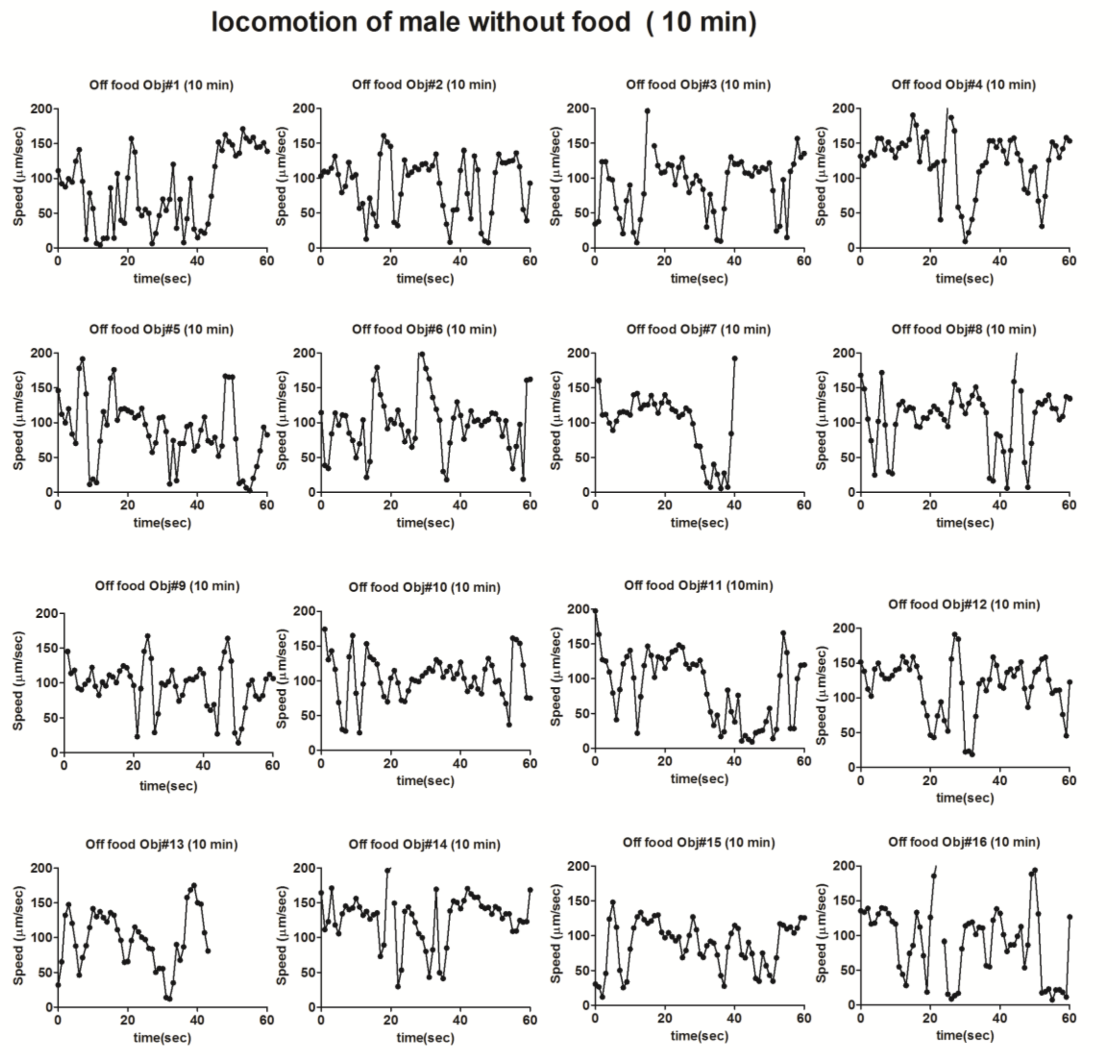

The locomotion of an individual male without food (10 min) shown in Figure S4b.

Supplementary Figure S7 (related to Fig. 8)

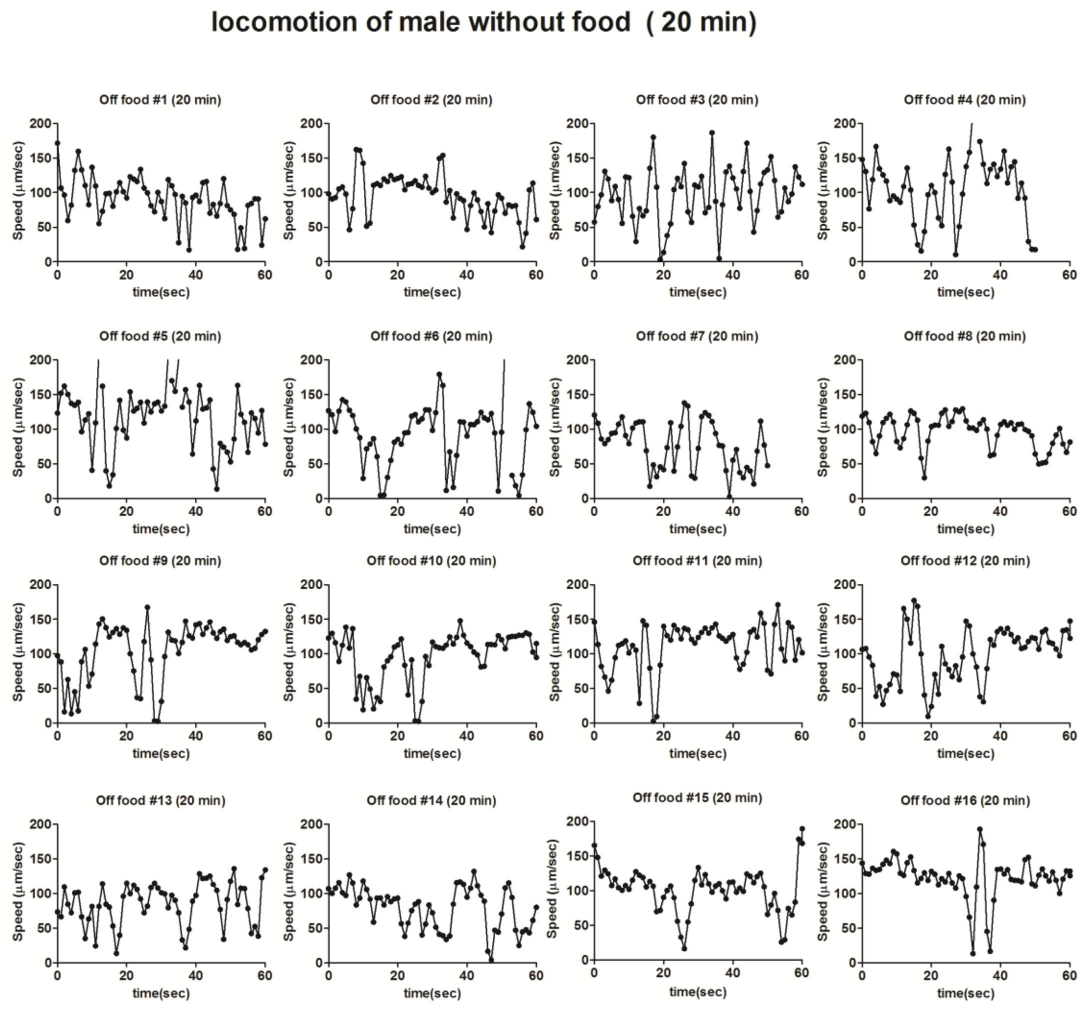

The locomotion of an individual male without food (20 min) shown in Figure S4c.

Supplementary Figure S8 (related to Fig. 8)

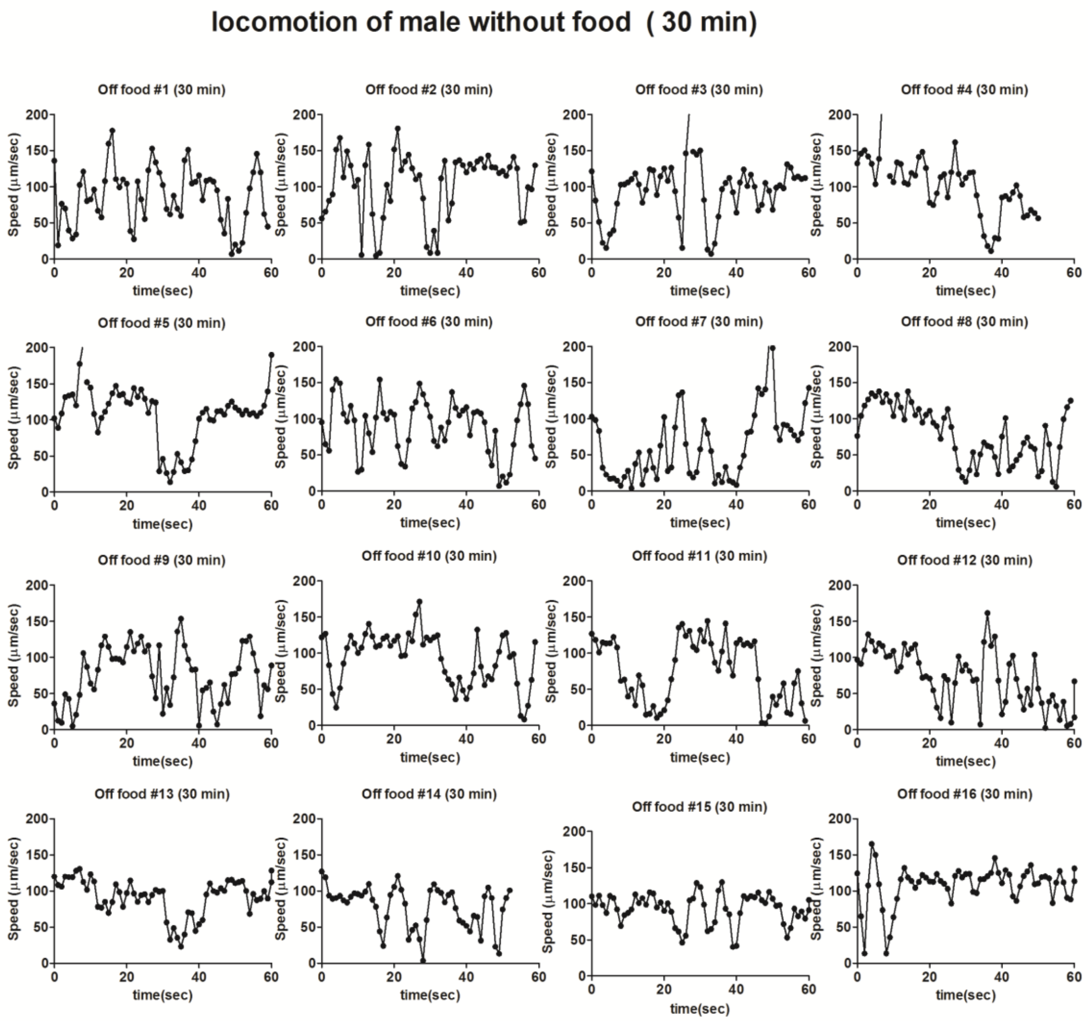

The locomotion of an individual male without food (30 min) shown in Figure S4d.

Supplementary Figure S9 (related to Fig. 8)

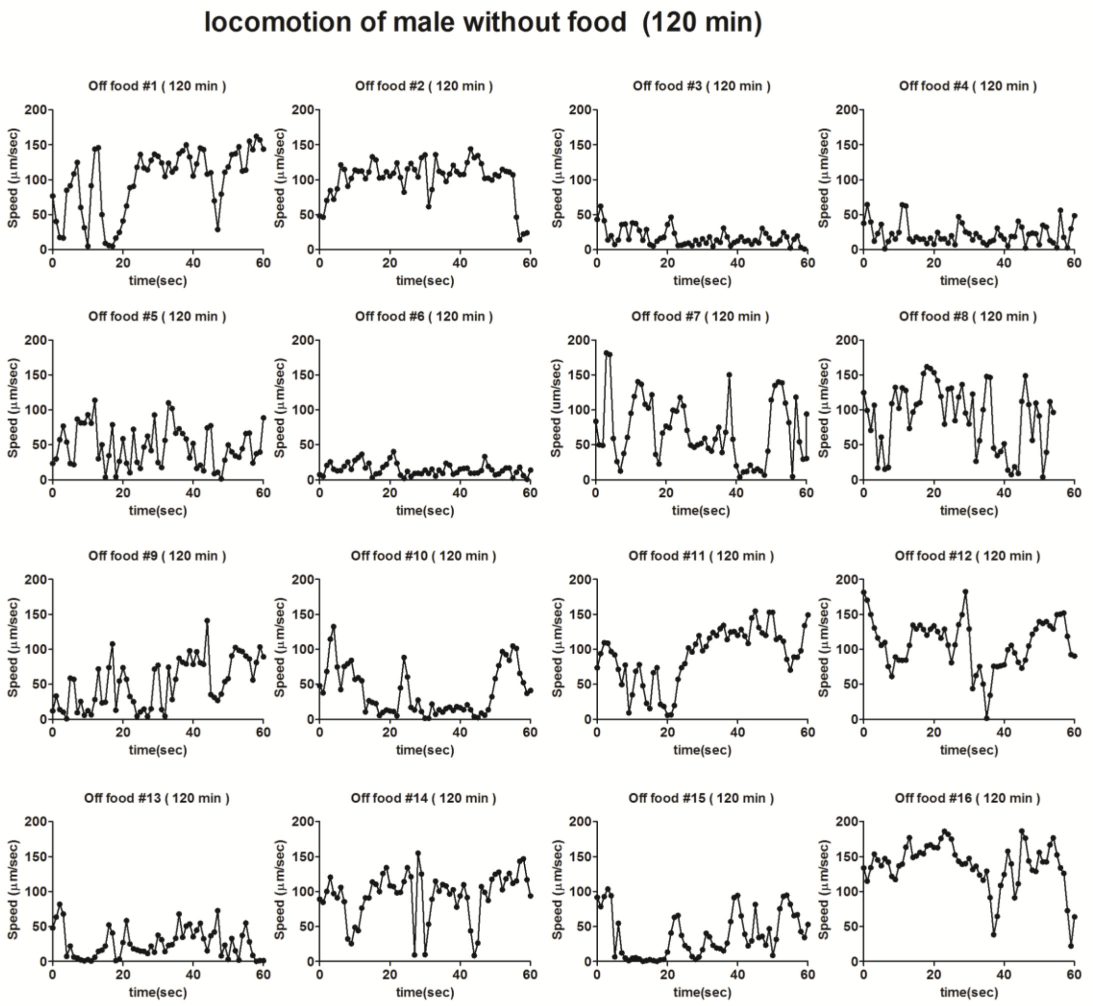

The locomotion of an individual male without food (120 min) shown in Figure S4e.

Supplementary Figure S10 (related to Fig. 5 and experimental procedures)

```
        10         20         30         40         50         60
atgacaagta ttatacgagc cgagtatgca gcctgccaag aattgaaaaa actggaaaac 60
agttcgtata atccgggagg ttgttcagtt gactttgaca aatcattatg ttgggcaagt 120
gcacatattg gacagcagat gacccgagac tgcccattca cattttgcac tgcaattcct 180
gggtgcgaag aaattaaaga cagatacatg gtttcccgga attgcaacag tatgggcgta 240
tggcaggact cgaattacac aatgtgcatc aaagttgttg aggaatatgc tcaatgtcta 300
caaggatttt gcagagtttg tcctgatgtt cttcgagact tggtgatatc ggtatcttta 360
acgttatcct ttgtatctgt catacttcta gtggcggcaa tcgttttgtt ttcgatattt 420
gactcgattc agtgccggag gttatcaatt cacaagaact tagcaactgc gttcgttttt 480
cgatttgctg ttttggcgat ttggacgatt gtgcaaacaa cgaacgtgtt tcaagattgt 540
acaagattaa ctccgctgcc tctttgggac tacgaatgga tttgcaaagc tattctctgg 600
ttcgtcatat atttcaacgt tgcgtccgtc atgtggatgt tgatcgaagg cgccttcctc 660
tacagtcgat tcactgtatt cgctatgcgt cacagtgatg caccatggtc tctttacctg 720
gcgtgtgggt ggggcgtccc gttcgtggtt gtaacggcat gggcattggt tcatcaatac 780
atatccagcc aacaaacaaa ttcattttgt tggctgcctt atgcccaggg actccatttg 840
tggattcttg ctggaactat gggatccgca ttaattatga atcttatatt cttgcttatg 900
attgtggtga tattggtgca aaagttacga acggagaatt ctgcgAaatc caagaaaatt 960
tggagaacaa taaaagcaac tcttttgtta gtgccactcc tcggcatttc aaacatccct 1020
ctttttacg agccggagca cccgagctct gtctacatgc tcggctcggc tattttacaa 1080
catagtcagg gtatttttat tgcggtcttg tattgcttct tgaatagtga aatccaggga 1140
gcgctgaagc ggcaattgtc aaaagtgcca tttgagttct tcaaaactag gaatcgattc 1200
gaaactgaaa gaacttacgt gcctgaagca agaaatgcca cgaaaaatgg agttccgatg 1260
gaggaaatga acaaaactaa aaatattgag agtggtgaaa atacggaatc tcaagatcag 1320
gttagcactg gaaagcaaat ctactcggtg tctacgaaat cttaa 1365
```

Sequences of pCJ61 (cDNA of *seb-3* including *eg696*). The red capital letter represents the *eg696* mutation (GtoA). pCJ122 contains WT cDNA of *seb-3*; G instead of A (shown in red).

Supplementary Table 1 (related to experimental procedures) list of Oligomers

primer	Seq.
pseb-3 F1	GGGGACAAGTTTGTACAAAAAAGCAGGCTccagttcaaattaactctacccaact ac
pseb-3 B1	GGGGACCACTTTGTACAAGAAAGCTGGGTtgagcaacaagtttctgaaaggttg
pseb-3 B2	GGGGACCACTTTGTACAAGAAAGCTGGGTtcattgagcaacaagtttctgaaagg
Infseb-3 F1	GGAGGACCCTTGAGGatgacaagtattatacgagccg
Infseb-3 B1	GAGGAGGCCATAGATTTCGTAGACACCGAGTAGAT
InfpGW F1	ATGGCCTCCTCCGAGGACGTC
InfpGW B1	CCTCAAGGGTCCTCCTGAAAATG
SDM1-F	cgtttttcgatttgctgttttggcgatttggacgattg
SDM1-B	aacgcagttgctaagttcttgtgaattgataacctccg
SDM2-F	gtgatgcaccatggtctctttacctggcg
SDM2-B	tgtgacgcatagcgaatacagtgaatcgactgtag
SDM3-F	gcttcttgaatagtgaaatccagggagcgc
SDM3-B	aatacaagaccgcaataaaaataccctgactatgttgtaaaatag
SDM4-F	cttttttacgagccggagcacccg
SDM4-B	agggatgtttgaaatgccgaggagtgg
SDM5-eg696-F	gttacgaacggagaattctgcgAaatccaag
SDM5-eg696-B	ttttgcaccaatatcaccacaatcataagcaagaata
HybIns-F1	ttcgtataatccggggtgggtc
HybIns-B1	tcaactgaacaacctctgaaatggca
HybVec-F1	aggttgttcagttgactttgacaaatcattatg
HybVec-B1	cccggattatacgaactgttttccagtt
attB-pgpa-10 F1	GGGGACAAGTTTGTACAAAAAAGCAGGCTCGTTCACAGTGTTTTCTA ACGTTTTTGC
attB-pgpa-10 B1	GGGGACCACTTTGTACAAGAAAGCTGGGTAGAAAGCTGGGTCCTGA ACATATATTGA
GCamp6-seb-3 Int.-F1	AGTGGAATAAGACAG gtgggtcaagttccagtgttc
GCamp6-seb-3 Int. B1	GCTCTGACTGCGTGAC ctgaaatggcataaatttaataattcctaatgatta
HybGCamp6-F1	GTCACGCAGTCAGAGCTATAGG
HybGCamp6-B1	CTGTCTTATTCCACTTACGACGTGAT

primer	Seq.
attB-5X QUAS-F1	GGGGACAAGTTTGTACAAAAAAGCAGGCTCCATGATTACGCCAAGCTTGC
attB-5X QUAS-B1	GGGGACCACTTTGTACAAGAAAGCTGGGTTTTGGGTCCTTTGGCCAATCC
134ins-F1	gtgtctacgaaatctATGAGTAAAGGAGAAGAACTTTTCACTGGAG
134ins-B1	TAGGAAACAGTTATGTTTGGTATATTGGGAATGTATTC
134vec-B1	AGATTTCGTAGACACCGAGTAGATTTGCTTTCCA
134vec-F1	CATAACTGTTTCCTACTAGTCGGCCGTACG
attBpgpa-10-F1	GGGGACAAGTTTGTACAAAAAAGCAGGCTCGTTCACAGTGTTTTCTAACGTTTTTGC
attBpgpa-10-B1	GGGGACCACTTTGTACAAGAAAGCTGGGTAGAAAGCTGGGTCCTGAACATATATTGA
attB-trx-1 F	GGGGACAAGTTTGTACAAAAAAGCAGGCT tctatattctgtctgaaattgaaccaattg
attB-trx-1 B	GGGGACCACTTTGTACAAGAAAGCTGGGTGATGAA ATACAAGTGTAG AAA ATT CAA ATA A
attB1 pklp-6 -F	GGGGACAAGTTTGTACAAAAAAGCAGGCTattcaccaaaaaattcattaaagcatt
attB2 pklp-6 -B	GGGGACCACTTTGTACAAGAAAGCTGGGTtattctgaaaagttcaactaataaa
